# *Kdm2a* deficiency in macrophages enhances thermogenesis to protect mice against HFD-induced obesity by enhancing H3K36me2 at the *Pparg* locus

**DOI:** 10.1038/s41418-020-00714-7

**Published:** 2021-01-18

**Authors:** Longmin Chen, Jing Zhang, Yuan Zou, Faxi Wang, Jingyi Li, Fei Sun, Xi Luo, Meng Zhang, Yanchao Guo, Qilin Yu, Ping Yang, Qing Zhou, Zhishui Chen, Huilan Zhang, Quan Gong, Jiajun Zhao, Decio L. Eizirik, Zhiguang Zhou, Fei Xiong, Shu Zhang, Cong-Yi Wang

**Affiliations:** 1 The Center for Biomedical Research, Department of Respiratory and Critical Care Medicine, NHC Key Laboratory of Respiratory Diseases, Tongji Hospital, Tongji Medical College, Huazhong University of Science and Technology, Wuhan, China; 2 Department of Nephrology,Tongji Hospital, Tongji College of Medicine, Huazhong University of Science and Technology, Wuhan, China; 3 Key Laboratory of Organ Transplantation, Ministry of Education, NHC Key Laboratory of Organ Transplantation, Key Laboratory of Organ Transplantation, Chinese Academy of Medical Sciences, Tongji Hospital, Wuhan, China; 4 Clinical Molecular Immunology Center, Department of Immunology, School of Medicine, Yangtze University, Jingzhou, China; 5 Department of Endocrinology and Metabolism, Shandong Provincial Hospital Affiliated to Shandong First Medical University, Jinan, China; 6 ULB Center for Diabetes Research, Université Libre de Bruxelles, 808 Route de Lennik, B-1070 Brussels, Belgium; 7 Diabetes Center, The Second Xiangya Hospital, Institute of Metabolism and Endocrinology, Central South University, Changsha, China

**Keywords:** Chronic inflammation, Histone post-translational modifications, Epigenetics, Endocrine system and metabolic diseases

## Abstract

Kdm2a catalyzes H3K36me2 demethylation to play an intriguing epigenetic regulatory role in cell proliferation, differentiation, and apoptosis. Herein we found that myeloid-specific knockout of *Kdm2a* (*LysM-*Cre-*Kdm2a*^f/f^, *Kdm2a*^*−/−*^) promoted macrophage M2 program by reprograming metabolic homeostasis through enhancing fatty acid uptake and lipolysis. *Kdm2a*^*−/−*^ increased H3K36me2 levels at the *Pparg* locus along with augmented chromatin accessibility and Stat6 recruitment, which rendered macrophages with preferential M2 polarization. Therefore, the *Kdm2a*^*−/−*^ mice were highly protected from high-fat diet (HFD)-induced obesity, insulin resistance, and hepatic steatosis, and featured by the reduced accumulation of adipose tissue macrophages and repressed chronic inflammation following HFD challenge. Particularly, *Kdm2a*^*−/−*^ macrophages provided a microenvironment in favor of thermogenesis. Upon HFD or cold challenge, the *Kdm2a*^*−/−*^ mice manifested higher capacity for inducing adipose browning and beiging to promote energy expenditure. Collectively, our findings demonstrate the importance of Kdm2a-mediated H3K36 demethylation in orchestrating macrophage polarization, providing novel insight that targeting Kdm2a in macrophages could be a viable therapeutic approach against obesity and insulin resistance.

## Introduction

Obesity arises from a complex interaction between genetic and environmental factors. Although genetic predisposition undoubtedly confers the risk of obesity, environmental exposures also play a crucial role in the initiation and progression of this disorder. Interestingly, epigenetic factors (e.g., DNA methylation and histone modification) serve as “fingerprints” to record gene-environment interactions or accumulated environmental exposures during the course of daily life processes [1]. Therefore, epigenetic factors have attracted growing interests as the mechanistic link between genetic variants and environmental factors in determining the risks of obesity [2]. In particular, histone modifications, such as acetylation, phosphorylation, ubiquitination, sumoylation, and methylation, regulate gene expressions by modulating chromatin structure [3]. Among which, histone methylation status is dynamically regulated by the lysine methyltransferases (KMTs) and lysine demethylases (KDMs). Kdm2a is a Jumonji C (JmjC) domain-containing histone KDM that catalyzes the demethylation of H3K36me2, which is associated with actively transcribed genes [4–8]. Kdm2a indeed has an intriguing epigenetic function since it is involved in a number of biological processes such as cell proliferation [9, 10], differentiation [11, 12], apoptosis [9], and tumorigenesis [7]. However, its role in obesity remains almost untouched.

It has now become evident that the immune system and metabolism are highly integrated, and obesity is characterized by the persistent and low-grade inflammation in the adipose tissue [13]. Particularly, in the lean adipose tissue, macrophages typically express “alternatively activated” (M2-like) markers and produce anti-inflammatory cytokines, contributing to the maintenance of tissue homeostasis, while “classically activated” (M1-like) macrophages that secrete pro-inflammatory mediators accumulate in the obese adipose tissue to promote insulin resistance [14, 15]. The differences of energy demands between M1 and M2 macrophages are emerging as regulatory circuits to regulate macrophage behavior. M1 macrophages depend on aerobic glycolysis for ATP production, but they suppress oxidative metabolism. Conversely, M2 macrophages prefer oxidative metabolism, especially fatty acid oxidation (FAO), as a mode of ATP generation [16, 17]. Other than metabolic status, epigenetic regulators are also critical to control the expression of genes essential for macrophage polarization by binding to their regulatory regions [17–19].

In the current study, we generated a macrophage-specific *Kdm2a*-deficient mouse model to dissect the impact of epigenetic regulation by Kdm2a on the development of obesity. Depletion of *Kdm2a* enhanced fatty acid uptake and the expression of oxidation network genes in a demethylase-dependent manner, thereby promoting macrophage M2 program. As a result, the mice were protected from high-fat diet (HFD)-induced obesity and insulin resistance. Mechanistically, Kdm2a demethylated H3K36me2 and remodeled chromatin accessibility on the *Pparg* locus. Our findings provided a novel insight into the understanding how epigenetic factors regulate macrophage programs implicated in the pathogenesis of obesity.

## Methods and materials

### Mice

The *Kdm2a*^flox/flox^ (*Kdm2a*^f/f^) mice in the C57BL/6 background were generated by targeting exons 7–9 (encoding the JmjC-catalytic domain) using a Cre-LoxP system. The Flp transgenic mice and *LysM*-Cre mice were purchased from the Jackson’s Laboratory (Bar Harbor, ME, USA). C57BL/6 mice were purchased from Beijing HFK Bioscience (Beijing, China). All mice were housed in a specific pathogen-free animal facility at the Tongji Medical College under a 12-h light/dark cycle with free access to food and water. Both male *LysM-*Cre*-Kdm2a*^f/f^ (referred as knockout (KO)) mice and their *Kdm2a*^f/f^ littermates (referred as wild-type (WT) controls) (8 weeks old) were either maintained on normal diet (ND, 9.4% kcal from fat; HFK Bioscience, Beijing, China) or switched to HFD (60% kcal from fat; Research Diet, Gardners, USA) for 16 consecutive weeks. Mice were allocated to ND group or HFD group randomly. For cold stimulation, the mice were chronically housed at 22 °C and acutely shifted to a 4–5 °C environment for 96 h in groups of two mice per cage. For controls at room temperature (RT), the mice were placed in a laboratory incubator at 22 °C. The core body temperature was recorded using a rectal probe. At the end of experiment, the mice were euthanized for subsequent biochemical experiments. All experimental procedures were approved by the Tongji Hospital Animal Care and Use Committee in accordance with the National Institutes of Health (NIH) guidelines.

### Human samples

Subcutaneous white adipose tissues (scWATs) from nonobese subjects (BMI: 18.5–24.9 kg/m^2^, *n* = 4) were obtained from intercostal fat during patients undergoing pinnaplasty. scWATs from obese patients (BMI ≥ 30 kg/m^2^, *n* = 4) were collected during bariatric surgery. All subjects were devoid of any evident systemic disease, chronic infection, or previous myocardial infarction. Smokers or patients undergoing thyroid hormone medication were excluded.

### Metabolic phenotyping

To analyze energy expenditure, single-housed mice were placed in metabolic cages connected with a comprehensive laboratory animal-monitoring system (Columbus Instruments, Columbus, OH). The mice were acclimatized to respiratory chambers for 48 h, followed by recording in real time for the data of oxygen consumption (VO_2_), carbon dioxide production (VCO_2_), respiratory exchange ratio (RER), and food intake. For glucose and insulin tolerance tests, the mice were peritoneally injected with glucose (Sigma-Aldrich Co., St. Louis, MO, USA) and insulin (Novolin R, Novo Nordisk Co., Bagsvaerd, Denmark) using the established techniques [20], and areas above curves were calculated for the ITT results as reported [21].

### Isolation of bone marrow-derived macrophages (BMDMs) and peritoneal macrophages

BMDMs were prepared as previously described [22]. Briefly, bone marrow was flushed from the tibiae and femurs of age-matched WT and KO mice. Bone marrow cells first underwent red blood cell lysis and were then resuspended in culture medium consisting of RPMI 1640, 10% fetal bovine serum (FBS), 1% penicillin/streptomycin (all from Gibco, Shanghai, China), and 30 ng/ml macrophage colony-stimulating factor (M-CSF, Peprotech, NJ, USA). On days 3 and 5 of differentiation, the media was replenished with fresh media containing 20 ng/ml M-CSF. On day 7, BMDMs were induced either by LPS (10 ng/ml, Sigma-Aldrich, St. Louis, MO) for M1 polarization, or by IL-4 (10 ng/ml, Peprotech, NJ, USA) for M2 polarization, as indicated duration. Peritoneal macrophages were obtained from male mice as previously reported [23, 24]. Briefly, the mice were intraperitoneally injected with 5 ml cold RPMI 1640. Peritoneal macrophages were harvested by washing peritoneal lavage twice with 5 ml cold RPMI 1640. After lysis of red blood cells, the cells were cultured in RPMI 1640 for 30 min to remove nonadherent cells. Adherent cells were then cultured in RPMI 1640 supplemented with 10% FBS, 1% penicillin/streptomycin, and recombinant IL-4 (10 ng/mL) as indicated time.

### Histological and morphological analysis

Samples from livers and adipose tissues were fixed with 4% paraformaldehyde for 24 h, embedded in paraffin, and then cut into 4-μm sections. After deparaffinization in xylene and rehydration in a graded alcohol series, all sections were subjected to H&E staining using the established techniques [25]. For immunohistochemical analysis, the antigens were retrieved in a citric acid solution (pH 6.0, 95–100 °C) for 20 min. The sections were next incubated with 3% H_2_O_2_ for 15 min, and then 5% goat serum for 1 h, followed by probing with a primary antibody against Ucp1 (R&D systems, Minneapolis, MN, USA) overnight at 4 °C. The sections were then developed using a Vector stain ABC kit (Vector Laboratories Inc., Burlingame, CA) and further counterstained with Harris’ hematoxylin as reported [25]. For immunostaining, rehydrated tissue sections were blocked with 5% donkey serum for 1 h at RT, and then incubated with di-methyl H3K36 (ab9049; Abcam, Cambridge, MA, USA), F4/80 (sc-377009; Santa Cruz Biotechnology, Santa Cruz, CA, USA), or CD68 (sc-17832; Santa Cruz Biotechnology, Santa Cruz, CA, USA) antibodies followed by staining with an AlexaFluor 488-conjugated anti-mouse IgG and an AlexaFluor 594-conjugated anti-rabbit IgG secondary antibody (Jackson ImmunoResearch Laboratories, West Grove, PA). Immunofluorescence signals were visualized under a fluorescence microscope (Leica, Wetzlar, Germany). Oil Red O staining of frozen liver sections was carried out using the previously described techniques [1]. Mean lipid droplet surface in H&E-stained adipose sections was quantified in a blinded fashion using the Image J 1.46r software (NIH, Wayne Rasband, USA). In all, 10–15 fields were analyzed for each sample by two examiners in a blinded fashion.

### Immunoblot analysis and antibodies

Tissues or cells were homogenized in the RIPA lysis buffer (Beyotime Biotechnology, Shanghai, China) containing the protease inhibitor cocktail (Roche, Indianapolis, IN). 40 μg of extracted proteins was separated by using 10% SDS-PAGE, and the separated proteins were transferred onto polyvinylidene difluoride membranes (Bio-Rad Laboratories, Hercules, Calif). The membranes were blocked with 5% nonfat milk for 1 h and then incubated with primary antibodies overnight at 4 °C. Antibodies against Kdm2a (ab191387) and di-methyl H3K36 (ab9049) were purchased from Abcam (Cambridge, MA, USA); antibodies against Arginase 1 (Arg1) (9819s), phospho-NF-κB p65 (Ser536) (3033s), IκBα (9242s), phospho-p38 MAPK (Thr180/Tyr182) (9216s), p38 MAPK (9212s), Pparγ (2443s), Stat6 (5397s), and NF-κB p65 (8242s) were ordered from Cell Signaling Technology (Danvers, MA, USA); antibodies against β-Actin (Sc-47778) and GAPDH (Sc-25778) were obtained from Santa Cruz Biotechnology (Santa Cruz, CA, USA); antibodies against CD36 (18836-1-AP) and Lamin B1 (12987-1-AP) were purchased from Proteintech (Wuhan, China); and antibodies against phospho-Stat6 (Tyr641) (abs130926) and tyrosine hydroxylase (TH, A0028) were originated from Absin (Shanghai, China) and Abclonal (Wuhan, China), respectively. After incubation with an HRP-conjugated secondary antibody, the reactive bands were visualized with an ECL Plus Western Blot Kit (Pierce, Rockford, Ill), and the relative intensity of reactive bands was analyzed using the Image J 1.46r software as previously described [26].

### ELISA

The concentrations of IL-6, IL-1β, and MCP-1 in serum were measured using the commercial ELISA kits obtained from Biolegend (San Diego, CA, USA), as previously described [23]. Serum insulin was determined using a Rat/Mouse Insulin ELISA kit (Millipore, Billerica, Merck KGaA, USA) as instructed.

### Real-time PCR

Total RNA extraction and cDNA synthesis were performed using the established techniques [27]. Real-time PCR was then carried out using an ABI prism 7500 Sequence Detection System (Applied Biosystems, CA, USA). PCR amplifications were carried out at 95 °C for 1 min, followed by 40 cycles at 95 °C for 15 s, 60 °C for 1 min. Relative expression levels for each target gene were calculated using the *2*^*−ΔΔCt*^ method. The primer sequences for real-time PCR are listed in Table S1.

### RNAi transfection

*Kdm2a* and *Cd36* were knocked down using the genOFF™ siRNA silencing kits (Ribobio, Guangzhou, China). We obtained RAW264.7 cells from ATCC. The cell line was routinely tested and authenticated negative for mycoplasma contamination. Cells cultured at about 60% confluency were transfected with RNAi oligonucleotides or negative control siRNA using the Lipofectamine™ 3000 Transfection Reagent (l3000-015; Invitrogen, Carlsbad, CA) in Gibco Opti-MEM reduced serum media. After washes following 18 h of transfection, the cells were resuspended in RPMI 1640 supplemented with 10% FBS and 1% penicillin/streptomycin, and ready for experimental purpose after 48 h of transfection. siRNA sequences were as follows: si-*Kdm2a*: 5′-CUA UGA GAC UCC AGA GGA A-3′; and si-*Cd36*: 5′-CAC AUA CAG AGU UCG UUA UUU-3′.

### Chromatin immunoprecipitation (ChIP) assay

ChIP assays were conducted using a ChIP assay kit (Beyotime Biotechnology, Shanghai, China), as previously described [28]. Briefly, the cells were harvested and cross-linked with 1% formaldehyde for 10 min at RT, followed by neutralizing with glycine for 5 min, and were then lysed in SDS lysis buffer. The cross-linked DNA was then sonicated with a UCD-300 Bioruptor (Diagenode, Denville, NJ, USA) to shell the chromatin to fragments ~200–1000 base pairs in length. The sonicated supernatants were precleared for 1 h with salmon sperm DNA/protein A + G agarose slurry at 4 °C, and then immunoprecipitated with antibodies against Stat6 (5397s; Cell Signaling Technology, Danvers, MA, USA) and H3K36me2 (ab9049; Abcam, Cambridge, MA, USA), or a control rabbit IgG (30000-0-AP; Proteintech, Wuhan, China) overnight. The salmon sperm DNA/protein A + G agarose beads (60 µl) were first incubated with sonicated supernatants for 1 h at 4 °C with rotation and then pelleted by centrifugation. The beads were next washed for 3–5 min with rotation in an order of low salt immune complex wash buffer, high salt immune complex wash buffer, LiCl complex wash buffer, and TE solution, respectively. The immune complexes were finally eluted out twice by addition of 250 μl of elution buffer (1% SDS, 0.1 M NaHCO3) by incubating 15 min with rotation at RT. The eluted DNA was then purified using a PCR Purification Kit (Qiagen, Redwood, CA, USA) and analyzed by quantitative PCR. Primer sequences used for ChIP assays are listed in Table S2.

### Flow cytometry

For surface markers, the cells were stained in PBS containing 1% BSA with indicated antibodies for 30 min on ice. For intracellular markers, the cells were first fixed with Fixation Buffer (420801; Biolegend, San Diego, CA, USA) at 4 °C for 30 min and then stained in Permeabilization Wash Buffer (421002; Biolegend, San Diego, CA, USA) with relevant antibodies at 4 °C for 30 min. Foxp3 staining was conducted according to the manufacturer’s instructions for the Mouse Foxp3 Buffer Set obtained from BD Bioscience (San Diego, CA, USA). The following antibodies were used for the studies: APC anti-mouse CD45 (103112), PE anti-mouse F4/80 (123110), PerCP/Cy5.5 anti-mouse F4/80 (123128), FITC anti-mouse CD11c (117306), APC anti-mouse CD206 (141708), APC anti-mouse/human CD45R/B220 (103211), PerCP/Cy5.5 anti-mouse Ly-6G/Ly-6C (108427), FITC anti-mouse CD4 (100406), PerCP anti-mouse CD8a (100732), AlexaFluor 647 anti-mouse/rat/human Foxp3 (320014), PE anti-mouse/human CD44 (103008), and APC anti-mouse CD62L (104412) from Biolegend (San Diego, CA, USA), and PE-Cy7 anti-mouse CD11b (552850) from BD Bioscience (San Diego, CA, USA). Flow cytometry data were acquired on MACSQuant^TM^ (Miltenyi Biotec, Auburn, CA, USA) and analyzed by FlowJo software (v10.5.3).

### Fatty acid uptake assay and seahorse assay

BODIPY (D3823; Invitrogen, Carlsbad, CA) powder was suspended in DMSO and aliquoted at 1 mg/ml. BMDMs were stimulated with 10 ng/mL IL-4 for 24 h, followed by starvation for 8 h. The starved cells were incubated with or without BODIPY (1:200 dilution) for 3 h at 37 °C, and its uptake was quantified by flow cytometry.

Basal oxygen consumption rate (OCR) was measured using a Seahorse XFe24 analyzer (Agilent Technologies, Santa Clara, CA, USA) according to the manufacturer’s instruction. In certain experiments, 5 μM GW9662 (a Pparγ inhibitor, HY-16578; MedChemExpress, Shanghai, China) was added into the cultures. BMDMs were stimulated with IL-4 for 24 h, and then seeded in an XF24 culture microplate (6 × 10^4^ cells/well) and pre-equilibrated for 1 h in the unbuffered XF assay medium supplemented with 25 mM glucose and 1 mM sodium pyruvate. Three or more consecutive measurements were obtained under basal conditions and after the sequential addition of compounds at the following final concentrations: 1 μM oligomycin, 1.5 μM FCCP, and 0.5 μM rotenone and antimycin A (all from Sigma-Aldrich, St. Louis, MO). In this assay, basal oxygen consumption can be determined by measuring OCR in the absence of drugs. Each condition was performed with 4–6 replicates, and the readings of OCR of each well were normalized to protein amount. XFe Wave software (Agilent Technologies, Santa Clara, CA, USA) was used to analyze the results.

### RNA sequencing (RNA-seq) and bioinformatic analysis

BMDMs were stimulated with or without IL-4 for 6 h, and mRNA was then extracted and subjected to deep RNA-seq analysis (BGI Genomics, Shenzhen, China). The prepared mRNA libraries were sequenced on an Illumina HISEQ 2500 (Illumina), and the Hisat2 v2.0.4 software (the Center for Computational Biology, Baltimore, Maryland) was used to map cleaned reads to the mm10 reference genome. Fragments per kilobase of exon per million mapped fragments (FPKMs) were calculated using the Cuffnorm version 2.2.1 software (University of Washington, Seattle, Washington, USA). The calculated genes with a normalized FPKM value > 1.0 were considered to be expressed. Significantly differentially expressed genes were defined as log2 (fold change) ≥ 0.5 with a *P* value ≤ 0.05. Heatmaps were generated using the R package pheatmap. The raw data have been deposited in the NCBI public repository Sequence Read Archive, with an accession code SRP234097.

### Assay for transposase-accessible chromatin with high-throughput sequencing (ATAC-seq) and data analysis

ATAC-seq was conducted in Frasergen (Wuhan, China) as previously reported [29] with minor modifications. Briefly, 5 × 10^4^ cells were resuspended in ice-cold nucleus lysis buffer (10 mM Tris pH 7.4, 10 mM NaCl, 3 mM MgCl2, and 0.1% IGEPAL CA-630) and centrifuged at 500 *×* *g* for 10 min at 4 °C. The supernatant was discarded, and the transposition reaction was performed using the Tn5 transposase at 37 °C for 30 min. Immediately following transposition, DNA was purified using a MinElute PCR Purification Kit (Qiagen, Redwood, CA, USA), and the resulting DNA was PCR amplified. The selection of size and purification of DNA fragments were done using the AMPure beads. Size distribution and molarity of the sequencing library were determined by Qubit (Thermo Fisher Scientific, Beverly, MA). Samples were performed in replicates and sequenced on an Illumina PE150 (Illumina) platform. Reads were mapped to the mm10 reference genome. Peaks with log2 (fold change) ≥ 0.5 and a *P* value ≤ 0.05 in comparisons were termed significant. Genome coverage (bedgraph) files were generated by the makeTagdirectory with checkGC parameter, and were used for visualization with IGV2. Read distribution (RD) around (RXR) peak summits were calculated within 51 × 30-nt bins by annotatePeaks.pl with -hist, -ghist options (HOMER; homer.ucsd.edu/homer/). RD plots were visualized by the Java TreeView, and histograms were visualized by the GraphPad Prism 5 software (GraphPad Software Inc., San Diego, CA). Analysis of DNA binding motif for transcription factors was computed using hypergeometric optimization of motif enrichment (HOMER; homer.ucsd.edu/homer/). The raw data have been deposited in the NCBI public repository Sequence Read Archive with an accession code SRP234183.

### Statistical analysis

All data were expressed as mean ± SEM, and all in vitro studies were performed at least three independent times with replications unless otherwise stated. Statistical analyses of the data were conducted with the GraphPad Prism 5 software (GraphPad Software Inc., San Diego, CA) using unpaired two-tailed Student’s *t* tests or one-way ANOVA where appropriate. For all statistical comparisons, differences with *P* values below 0.05 were considered statistically significant. Detailed approaches for bioinformatic analysis of RNA-seq and ATAC-seq data were described above.

## Results

### Obesity is featured by the reduced H3K36me2 levels in adipose tissue macrophages (ATMs)

**Fig. 1:**
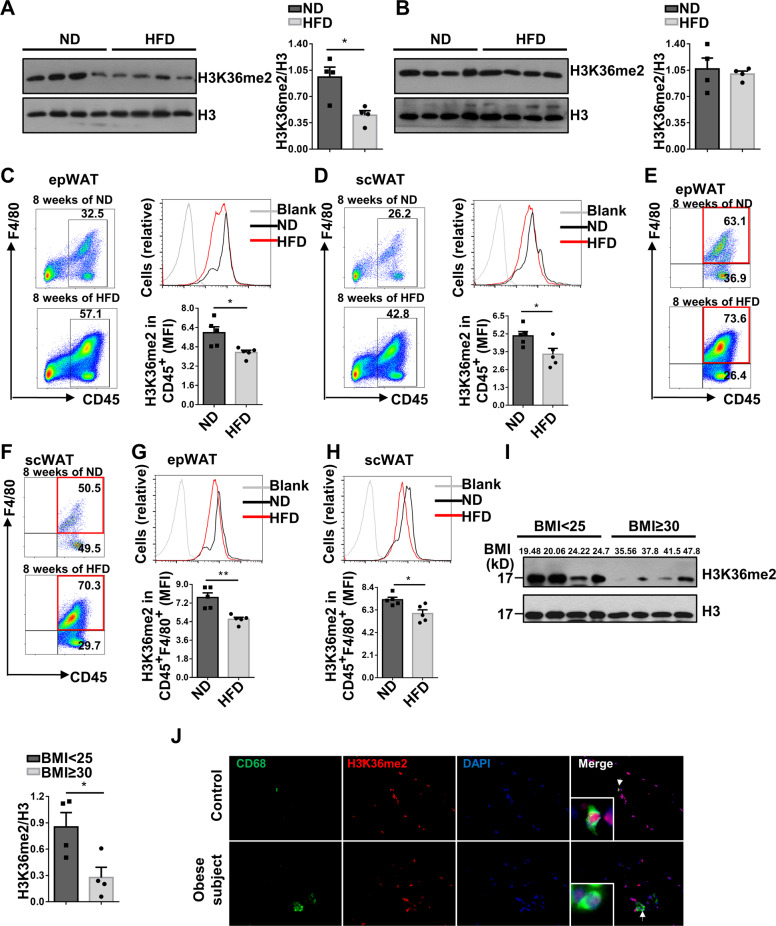
Obesity is featured by the reduced H3K36me2 levels in ATMs. H3K36me2 levels in epWAT (**A**) and scWAT (**B**) of mice fed ND or HFD for 8 weeks (*n* = 4/group). Flow cytometry analysis of H3K36me2 status in CD45^+^ cells from epWAT (**C**) and scWAT (**D**) of mice fed with ND or HFD for 8 weeks. Left: a representative flow cytometry data; right: quantitative data from all mice analyzed (*n* = 5/group). Representative FACS plots showing the proportion of F4/80^+^ cells gated on CD45^+^ cells from epWAT (**E**) and scWAT (**F**) of mice fed with ND or HFD for 8 weeks. Representative FACS plots and quantitative data of H3K36me2 levels in macrophages from epWAT (**G**) and scWAT (**H**) of mice after 8 weeks of HFD or ND feeding (*n* = 5/group). **I** Western blot analysis of H3K36me2 levels in CD45^+^ cells isolated from scWAT of controls (*n* = 4) and obese subjects (*n* = 4). **J** Representative immunostaining results for CD68 and H3K36me2 in scWAT sections from controls and obese subjects. Scale bars: 50 μm. Original magnification: ×400. Values are expressed as mean ± SEM, and unpaired Student’s *t* test was employed for data analysis. *P < 0.05; **P < 0.01.

We first analyzed H3K36me2 levels in epididymal white adipose tissues (epWATs) and scWATs from C57BL/6 mice fed following ND or HFD challenge. epWAT from HFD-induced mice manifested significantly decreased H3K36me2 levels (Fig. 1A) but not in scWAT (Fig. 1B). Next, stromal vascular fraction including both CD45^−^ and CD45^+^ cells was isolated from epWAT, and no significant difference of H3K36me2 levels in CD45^−^ cells between ND and HFD-fed mice was detected (Fig. S1A, B). However, the infiltrated CD45^+^ immune cells manifested a significantly decreased H3K36me2 levels (Fig. 1C), and a similar trend was noted in CD45^+^ cells from scWAT from obese mice (Fig. 1D). To define the cell type that causes differential H3K36me2 levels, we embarked on ATMs as they are the predominant cell type in CD45^+^ cells (Fig. 1E, F). Indeed, significantly lower levels of H3K36me2 were noted in ATMs from both epWAT (Fig. 1G) and scWAT (Fig. 1H) of obese mice. Since no significant difference in terms of H3K36me2 levels in scWAT between obese and control mice was detected, this discrepancy was likely caused by the lower number of ATMs in scWAT. However, the reduction in H3K36me2 levels after HFD feeding was not observed in splenic CD45^+^ cells (Fig. S1C) and macrophages (Fig. S1D), indicating that this phenomenon was not a general event.

We further confirmed the above observations in obese patients. Consistently, adipose CD45^+^ cells from obese subjects displayed significantly lower levels of H3K36me2 (Fig. 1I). To demonstrate whether the difference of H3K36me2 levels occurred in ATMs as observed in obese mice, we conducted co-immunostaining of CD68 (a marker for human macrophages) and H3K36me2. Indeed, much lower levels of H3K36me2 fluorescent intensity were noted in adipose section originated from obese subjects (Fig. 1J). Collectively, our data suggest that obesity is characterized by the decreased H3K36me2 levels in ATMs.

### *Kdm2a* deficiency augments the production of M2 macrophages

**Fig. 2:**
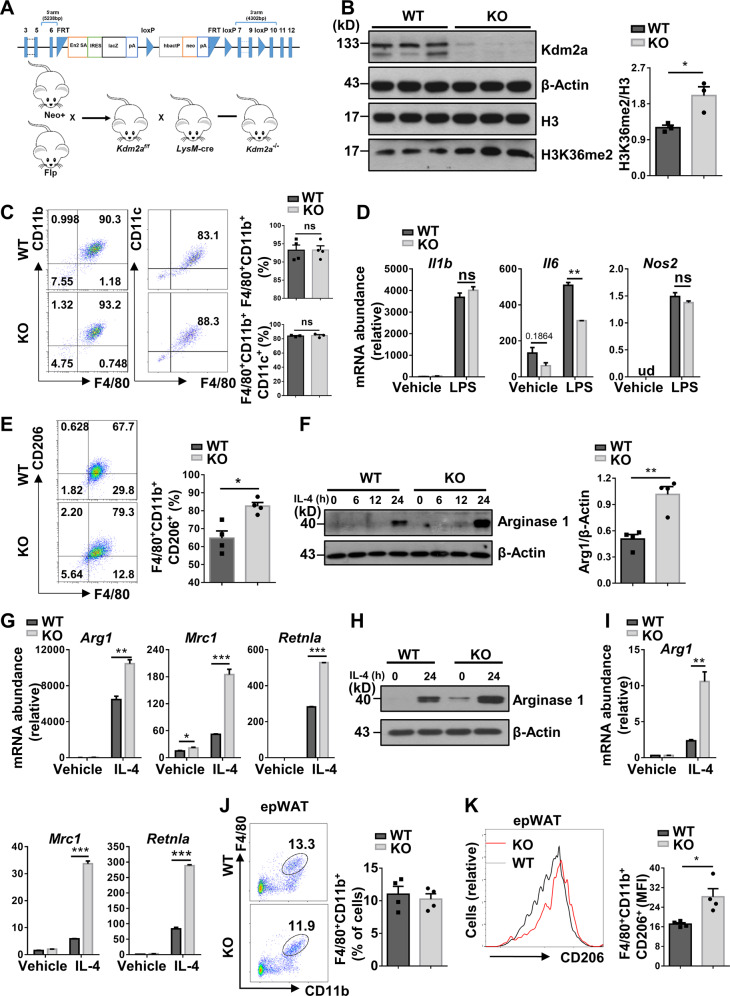
*Kdm2a* deficiency orchestrates alternative activation of macrophages. **A** Strategy for generating a conditional *Kdm2a*-deficient model. *Kdm2a* exons 7*–*9 were flanked by a neo-flippase recognition target (FRT) and two loxP sites. The neo-gene was deleted after Flp recombination and the *Kdm2a* exons were then excised by Cre recombinase. **B** Western blot analysis of Kdm2a and H3K36me2 levels in BMDMs from WT and KO mice, and a bar graph showing data derived from three mice in each group. **C** Flow cytometry analysis of the expression of F4/80, CD11b, and CD11c in BMDMs stimulated with LPS for 24 h. The percentages of F4/80^+^CD11b^+^ and F4/80^+^CD11b^+^CD11c^+^ cells are shown. **D** RT-PCR analysis of relative mRNA expression of M1 marker genes in BMDMs stimulated with LPS for 6 h. **E** Flow cytometry analysis of the expression of F4/80, CD11b, and CD206 in BMDMs stimulated with IL-4 for 24 h. Percentages of F4/80^+^CD11b^+^CD206^+^ cells are shown in the bar graphic figure. **F** Western blot analysis of Arg1 expression in BMDMs stimulated with IL-4 with indicated time. **G** RT-PCR analysis of relative mRNA levels of M2 genes in BMDMs stimulated with IL-4 for 6 h. **H** Western blot analysis of Arg1 expression in peritoneal macrophages with or without IL-4 stimulation for 24 h. **I** RT-PCR analysis of relative mRNA levels of M2 genes in peritoneal macrophages stimulated with or without IL-4 for 6 h. **J** Representative FACS plots and the percentages of F4/80^+^CD11b^+^ cells in epWAT of ND-fed WT and KO mice at 16 weeks of age (*n* = 4/group). **K** Expression of CD206 in F4/80^+^CD11b^+^ cells as shown in (**J**). The amounts of F4/80^+^CD11b^+^CD206^+^ cells were quantified and shown as relative mean fluorescence intensity (MFI). Results are representative of three to four independent experiments (**B**–**I**). Values are expressed as mean ± SEM, and unpaired Student’s *t* test was used for data analysis. *P < 0.05; **P < 0.01; ***P < 0.001. ns not significant, ud undetected, Arg1 Arginase 1.

Given that Kdm2a catalyzes the demethylation of H3K36me2, we generated a macrophage-specific *Kdm2a* KO model using the strategy shown in Fig. 2A and *Kdm2a* deficiency was genotyped by PCR analysis (Fig. S2A). Western blot analysis confirmed that Kdm2a protein expression was abolished in BMDMs (Fig. 2B) but remained normal in CD4^+^ T cells as well as in adipose tissues and liver (Fig. S2B). Loss of *Kdm2a* resulted in a significant increase of H3K36me2 levels in macrophages (Fig. 2B), which did not lead to a compensated *Kdm2b* overexpression that also targets H3K36 (Fig. S2C). The KO mice survived and grew normally, and produced similar number of neutrophils, macrophages, and dendritic cells in the bone marrow and spleen as their *Kdm2a*^f/f^ littermates (referred as WT controls), indicating that the development and maturation of myeloid cells appeared to be normal (Fig. S3A–F). Moreover, loss of *Kdm2a* did not influence the maturation and activation of lymphoid cells in the periphery (Fig. S3G–M).

Next, BMDMs were generated from 8-week-old KO mice and littermates, followed by LPS or IL-4 induction as described. Interestingly, *Kdm2a* deficiency did not affect BMDM generation and M1 program (F4/80^+^/CD11b^+^/CD11c^+^) (Fig. 2C), but *Kdm2a*^*−/−*^ BMDMs manifested downregulated *Il6* expression following LPS induction, while the M1 program-related genes such as *Il1b* and *Nos2* were unaltered (Fig. 2D). As NF-κB and MAPK signaling are critical for IL-6 production in response to LPS stimulation, we then examined their upstream essential molecules, but failed to detect a perceptible difference (Fig. S4A). In sharp contrast, *Kdm2a* deficiency significantly promoted macrophage M2 program upon IL-4 induction as evidenced by the higher number of CD206^+^ cells (Fig. 2E), although no discernable difference of CD206^+^ cells was observed before IL-4 induction (Fig. S4B). Western blot analysis also confirmed enhanced Arg1 expression, another marker for M2 macrophages (Fig. 2F), and RT-PCR analysis detected significantly increased mRNA levels for M2 markers such as *Arg1*, *Mrc1*, and *Retnla* (Fig. 2G). Consistently, the enhanced M2 program was also noted in the KO peritoneal macrophages following IL-4 stimulation (Fig. 2H, I). Moreover, comparable amounts of total macrophages (F4/80^+^/CD11b^+^) (Fig. 2J) but considerably increased M2 macrophages (F4/80^+^/CD11b^+^/CD206^+^) in the epWAT were noted from KO mice as compared with their littermates (Fig. 2K) once they fed with ND. Together, those data support that Kdm2a selectively orchestrates macrophage M2 program.

### *Kdm2a*^*−/−*^ macrophages display enhanced fatty acid uptake along with metabolic reprogramming

**Fig. 3:**
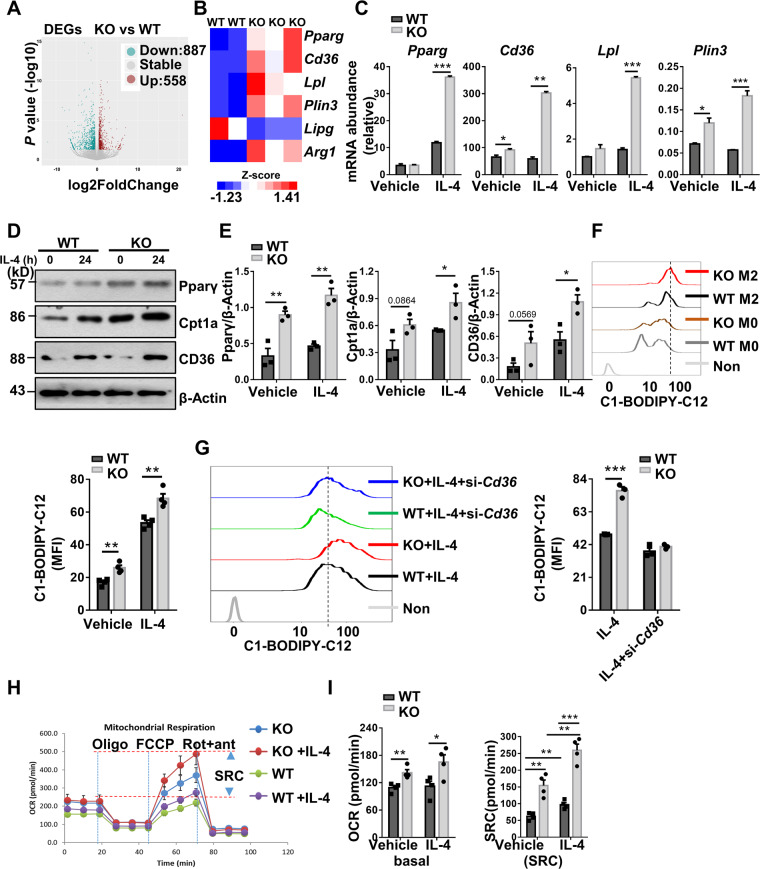
Loss of *Kdm2a* in myeloid cells upregulates Pparγ and promotes fatty acid uptake and oxidation. **A** A scatter plot representing the average gene expression levels (−log10) in KO relative to WT BMDMs upon IL-4 stimulation vs. fold changes (log2). Two biological replicates for WT mice and three for KO mice were included, and there were two mice for each biological replicate. **B** Heatmap showing the differentially expressed genes relevant to M2 program and genes related to lipid metabolism in IL-4-treated BMDMs. The data were generated from RNA deep sequencing as described. **C** RT-PCR analysis to confirm the relative mRNA abundance of *Pparg*, *Cd36*, *Lpl*, and *Plin3* in BMDMs treated with vehicle or IL-4 for 6 h. Representative western blot results for analysis of Pparγ, CD36, and Cpt1a expression in BMDMs before and after IL-4 stimulation (**D**), and bar graphs (**E**) showing the mean expression levels resulted from three independent replications. **F** Uptake of BODIPY-labeled C12-fatty acids in BMDMs treated with or without IL-4 by flow cytometry analysis, and a bar graphic figure showing the mean data derived from four independent replications. **G** Representative FACS plots and quantitative data showing the uptake of BODIPY-labeled C12-fatty acids in BMDMs under indicated conditions. **H** OCR of BMDMs derived from WT and KO mice following 24 h of IL-4 stimulation, which was assessed before and after sequential treatment with oligomycin, FCCP, rotenone, and antimycin A. **I** Basal OCR (left) and SRC (right) in cells shown in (**H**), and all data were presented as mean derived from four independent experiments. The values are presented as mean ± SEM. Significance was determined by unpaired Student’s *t* test in (**C**, **E**–**G**, **I**) (left panel) and by one-way ANOVA in (**I**) (right panel). *P < 0.05; **P < 0.01; ***P < 0.001. Cpt1a carnitine palmitoyltransferase 1a.

Since both BMDMs and ATMs from the KO mice displayed a similar phenotype and limited ATM number, while bone marrow has been identified as an important contributor to the ATM pool [30, 31], we used BMDMs for further analyses. To get insight into the mechanisms by which Kdm2a regulates macrophage M2 program, RNA deep sequencing was conducted. As compared to WT BMDMs, the KO BMDMs are characterized by the 558 upregulated and 887 downregulated genes (Fig. 3A). Interestingly, only a few transcriptional factors relevant to macrophage polarization displayed altered expressions, and among which, *Pparg* was noted to be significantly upregulated in the KO macrophages along with a marked reduction of *Irf1* and *Hif1a* (Fig. S4C). We then analyzed the RNA-seq data with focus on the genes relevant to M2 program. Other than *Pparg* those genes relevant to lipid metabolism such as *Cd36*, *Lpl*, and *Plin3* were also significantly upregulated in the KO cells (Fig. 3B), which were further confirmed by RT-PCR analysis (Fig. 3C). Indeed, western blot analysis of above lipid metabolism-associated proteins, including Cpt1a, a critical Pparγ downstream molecule in BMDM lysates revealed that IL-4 induced significantly higher levels of Pparγ, Cpt1α, and Cd36 protein expression in KO BMDMs (Fig. 3D, E).

The above data prompted us to assume that *Kdm2a* deficiency promotes macrophage M2 program by regulating metabolic reprogramming. We thus first examined lipid uptake using BODIPY-labeled C12-fatty acids. The KO BMDMs displayed significantly higher capacity to take fatty acids than WT BMDMs (Fig. 3F), which was CD36-dependent as knockdown of CD36 rescued this discrepancy (Fig. 3G). We then monitored oxygen consumption as described, and the KO BMDMs exhibited markedly higher basal OCR along with increased spare respiratory capacity (SRC) in response to either vehicle or IL-4 stimulation (Fig. 3H, I), indicating that the KO BMDMs are characterized by the increased commitment to oxidative phosphorylation (OXPHOS), which favors macrophage M2 program [32]. Altogether, these results suggest that *Kdm2a* deficiency promotes fatty acid uptake associated with metabolic reprogramming, thereby enhancing macrophage M2 program.

### Mice with macrophage *Kdm2a*^*−/−*^ are protected from HFD-induced obesity and insulin resistance

**Fig. 4:**
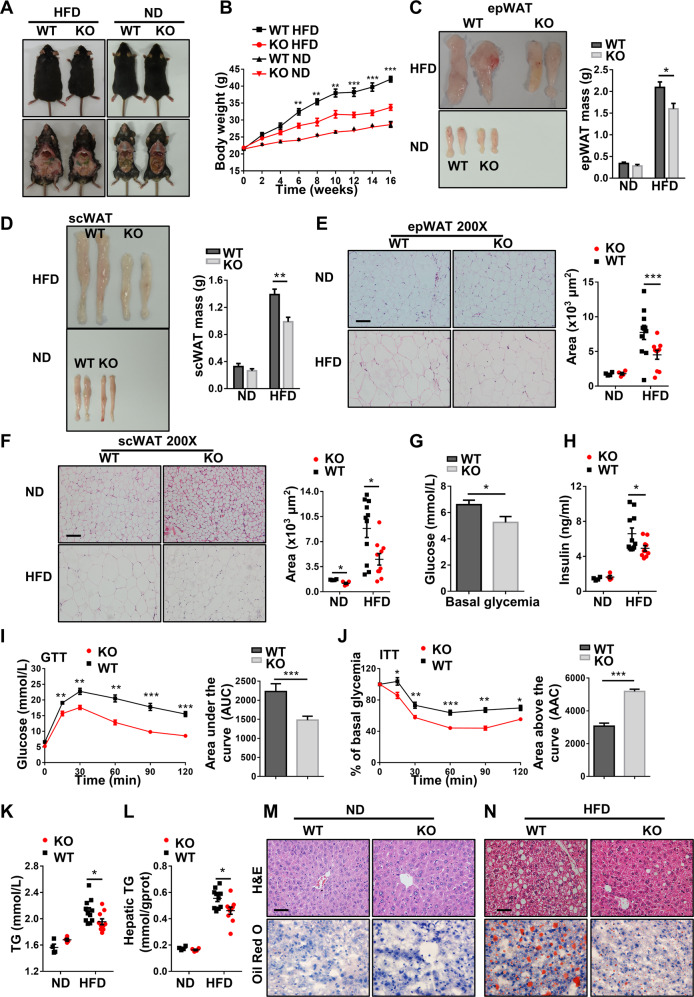
Loss of *Kdm2a* alleviates HFD-induced obesity and obesity-associated metabolic deterioration. **A** Representative images for WT and KO mice fed with HFD or ND for 16 weeks. **B** Body weight changes for WT and KO mice during the course of HFD or ND induction. Representative pictures and bar graphs of epWAT (**C**) and scWAT (**D**) mass collected from WT and KO mice after 16 weeks of HFD or ND feeding. Representative H&E staining and lipid droplet surface quantification of epWAT (**E**) and scWAT (**F**) from HFD- or ND-fed WT and KO mice. **G** Blood glucose levels between WT and KO mice under fasting condition. **H** Plasma insulin levels determined after 16 weeks of HFD or ND feeding. **I** Results of intraperitoneal glucose tolerance tests (left) and the areas under curves (AUC) for blood glucose levels (right). **J** Results for the intraperitoneal insulin tolerance tests and areas above curves (AAC). **K** Results for analysis of triglyceride (TG) levels in the plasma. **L** Results for TG levels in the liver. Representative H&E staining and Oil Red O staining of liver sections originated from ND (**M**) or HFD (**N**) fed WT and KO mice. ND, *n* = 4 per group; HFD, *n* = 11 for WT mice and *n* = 10 for KO mice. Scale bars: 100 μm (**E**, **F**); 50μm (**M**, **N**). Original magnification: ×200 (**E**, **F**); ×400 (**M**, **N**). Values are expressed as mean ± SEM and unpaired Student’s *t* test was employed for data analysis. *P < 0.05; **P < 0.01; ***P < 0.001.

Since ATMs play a central role in the pathogenesis of obesity and insulin resistance [13], we thus fed 8-week-old KO mice and WT littermates with HFD for 16 weeks. Remarkably, the KO mice were significantly protected from HFD-induced obesity, as evidenced by the significantly lower body weight (Fig. 4A, B) along with a marked reduction of the WAT mass (Fig. 4C, D) and smaller sizes of adipocytes (Fig. 4E, F).Consistently, the KO mice displayed lower basal glucose levels than WT littermates after HFD induction (Figs. 4G and S5A). Similarly, the KO mice showed decreased serum insulin levels (Fig. 4H) along with significantly improved glucose tolerance (Figs. 4I and S5B) and insulin sensitivity (Fig. 4J). Even under ND, the KO mice showed substantially higher insulin sensitivity (Fig. S5C). The KO mice were also featured by the significantly lower levels of plasma (Fig. 4K) and hepatic triglycerides (Fig. 4L) coupled with a reduced intrahepatic lipid accumulation as determined by H&E and Oil Red O staining (Fig. 4M, N). Together, our data support that mice with macrophage *Kdm2a*^*−/−*^ are protected from HFD-induced obesity, insulin resistance, and hepatic steatosis.

### Loss of *Kdm2a* reduces ATM accumulation and represses chronic inflammation in the adipose tissue following HFD challenge

**Fig. 5:**
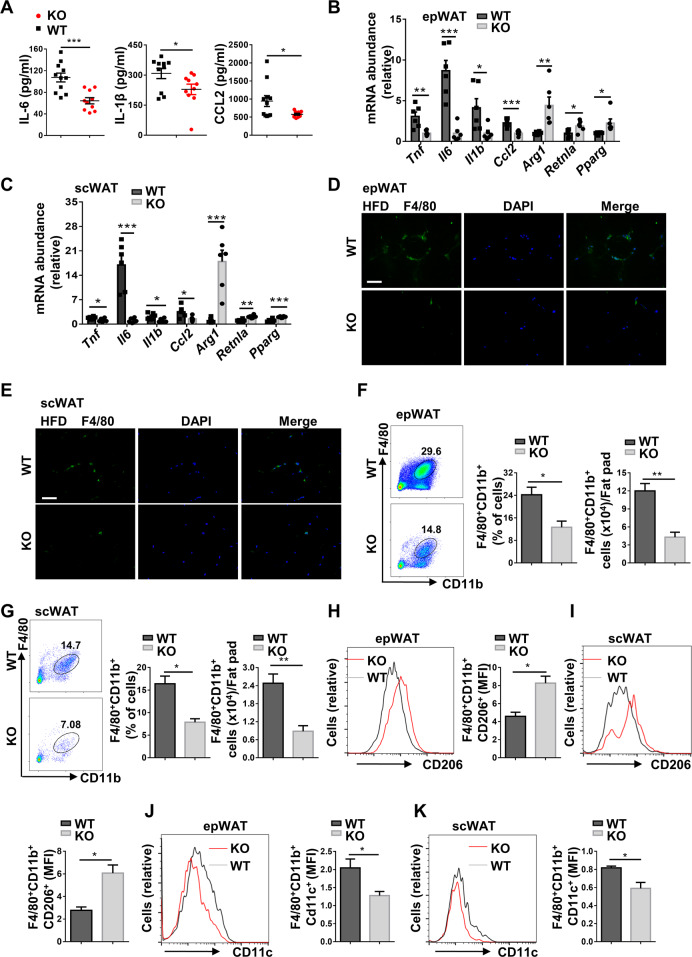
*Kdm2a* deficiency alleviates macrophage accumulation and reverses the M1–M2 imbalance of ATMs in mice following HFD challenge. **A** Analysis of plasma cytokine levels between HFD-induced WT (*n* = 11) and KO (*n* = 10) mice. RT-PCR analyses of the mRNA abundance for the indicated genes in epWAT (**B**) and scWAT (**C**) (*n* = 6 per group). Representative images of immunostaining for F4/80 in epWAT (**D**) and scWAT (**E**) sections. The images were taken under ×400 magnification. Scale bar: 50 μm. Representative FACS plots, and the frequency and the number of ATMs in epWAT (**F**) and scWAT (**G**) in HFD-induced WT and KO mice. Representative FACS plots and quantitative data of M2 macrophages in epWAT (**H**) and scWAT (**I**) from HFD-induced WT and KO mice. Flow cytometry analysis of M1 macrophages in epWAT (**J**) and scWAT (**K**) from HFD-induced WT and KO mice. Left: a representative plot for flow cytometry; right: quantitative data resulted from all mice analyzed. *n* = 4 for each group (**F**–**K**). Values are presented as mean ± SEM, and unpaired Student’s *t* test was used for statistical analysis. *P < 0.05; **P < 0.01; ***P < 0.001.

We next sought to address the effect of *Kdm2a* deficiency on HFD-induced chronic inflammation in the adipose tissues. We first detected markedly lower levels of serum IL-6, IL-1β, and CCL2 in the HFD-induced KO mice (Fig. 5A). Indeed, both epWATs (Fig. 5B) and scWATs (Fig. 5C) displayed significantly lower levels of *Tnf*, *Il6*, *Il1b*, and *Ccl2* expression, but higher levels of expression for anti-inflammatory markers such as *Arg1*, *Retnla*, and *Pparg* by RT-PCR analysis. Immunostaining of WAT sections indicated lower numbers of cells with F4/80^+^ crown-like structures in HFD-induced KO mice (Fig. 5D, E), and less accumulation of total macrophages was further confirmed by flow cytometry in the epWATs (Fig. 5F) and scWATs (Fig. 5G). Compared with KO mice, the H3K36me2 levels in ATMs of WT mice were further decreased following HFD induction (Fig. S5D, E), which was consistent with the data shown in Fig. 1. A significantly higher proportion of M2 macrophages was detected both in epWATs (Fig. 5H) and scWATs (Fig. 5I) from KO mice, while the WT mice showed a much higher proportion of M1 macrophages both in epWATs (Fig. 5J) and scWATs (Fig. 5K). Collectively, those results demonstrate that Kdm2a^*−/−*^ attenuates HFD-induced ATM accumulation coupled with a higher M2 proportion.

### *Kdm2a*^*−/−*^ promotes energy expenditure and adipose tissue browning

**Fig. 6:**
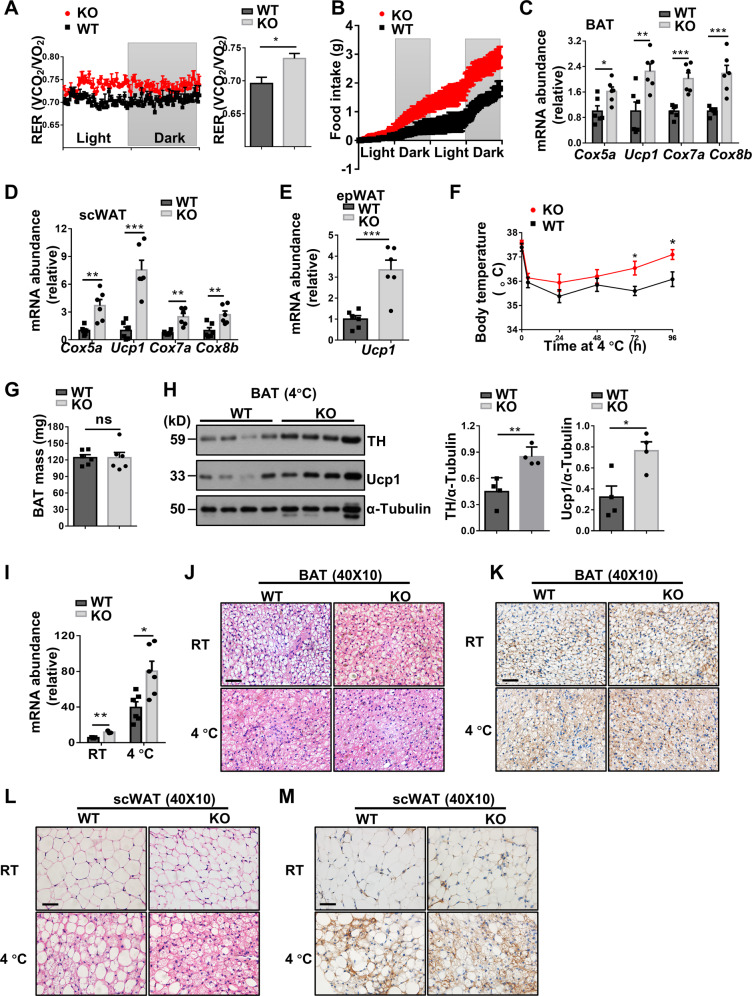
Macrophages deficient in *Kdm2a* increase energy expenditure and adaptive thermogenesis. **A** Results for real-time recording of RER (left) and quantitative data (right) collected from four mice in each group for 24 h following 16 weeks of HFD induction (*n* = 4 per group). **B** Food intake (g) in WT and KO mice following 16 weeks of HFD induction measured in metabolic cages (*n* = 4 per group). RT-PCR results for analysis of thermogenic genes *Cox5a*, *Ucp1*, *Cox7a*, and *Cox8b* in BAT (**C**) and scWAT (**D**) from HFD challenged WT and KO mice (*n* = 6 for each group). **E** RT-PCR results for analysis of *Ucp1* in the epWAT following 16 weeks of HFD challenge (*n* = 6 for each group). **F** Daily rectal temperature of mice housed under 4 °C condition (*n* = 6 per group). **G** Analysis of the weight for BAT collected from WT and KO mice after cold exposure (*n* = 6 per group). **H** Representative western blot results (left) and bar graphs (right) showing the expression levels for BAT TH and Ucp1 (*n* = 4 per group). **I** Relative mRNA abundance of *Ucp1* in BAT of mice with (*n* = 6/group) or without cold stress (*n* = 3/group). Representative images of H&E staining (**J**) and Ucp1 IHC (**K**) of BAT sections with or without cold exposure (six images per mouse). Representative images of H&E staining (**L**) and Ucp1 IHC (**M**) of scWAT sections (six images per mouse). Scale bars: 50 μm (**J**–**M**). Original magnification: ×400 (**J**–**M**). Values are expressed as mean ± SEM, and unpaired Student’s *t* test was used for data analysis. *P < 0.05; **P < 0.01; ***P < 0.001. ns not significant.

To further address whether Kdm2a^*−/−*^ in macrophages influences energy homeostasis, the mice were subjected to analysis of metabolic indexes. The KO mice were characterized by the significantly higher RER and food consumption than control mice following HFD challenge (Fig. 6A, B), but they manifested comparable RER and food intake as control mice under ND (Fig. S5F, G). Next, we examined the expression of thermogenic genes by real-time PCR. Substantially higher levels of *Cox5a*, Ucp1, Cox7a, and *Cox8b* mRNA were detected in the brown adipose tissue (BAT, Fig. 6C) and scWAT (Fig. 6D) of KO mice following HFD induction, and the KO mice displayed a 2.2-fold higher *Ucp1* expression in the epWAT than that of WT mice (Fig. 6E).

The above results prompted us to examine the impact of *Kdm2a*^*−/−*^ on adaptive thermogenesis. The KO mice maintained their body temperatures at higher levels than controls after cold exposure (4–5 °C) for 4 days (Fig. 6F), although no perceptible change was noted in terms of BAT weight (Fig. 6G). Immunoblotting indicated a marked upregulation of TH and Ucp1 in the BAT of KO mice following cold induction (Fig. 6H). RT-PCR analysis further revealed that BAT originated from KO mice had higher levels of *Ucp1* mRNA, both at RT and following cold exposure (Fig. 6I). Consistently, the potency for cold-induced decrease of lipid droplet levels (Fig. 6J) and increase of Ucp1 expression (Fig. 6K) in the BAT was much higher in the KO mice. Furthermore, scWAT derived from cold-induced KO mice exhibited markedly enhanced remodeling (Fig. 6L) along with increased induction of multilocular Ucp1-expressing beige adipocytes (Fig. 6M). Collectively, our data suggest that macrophage deficient in *Kdm2a* provides a microenvironment in favor of thermogenesis by inducing adipose browning and beiging.

### Kdm2a demethylates H3K36me2 at the *Pparg* locus and blunts the recruitment of Stat6 to *Pparg*

**Fig. 7:**
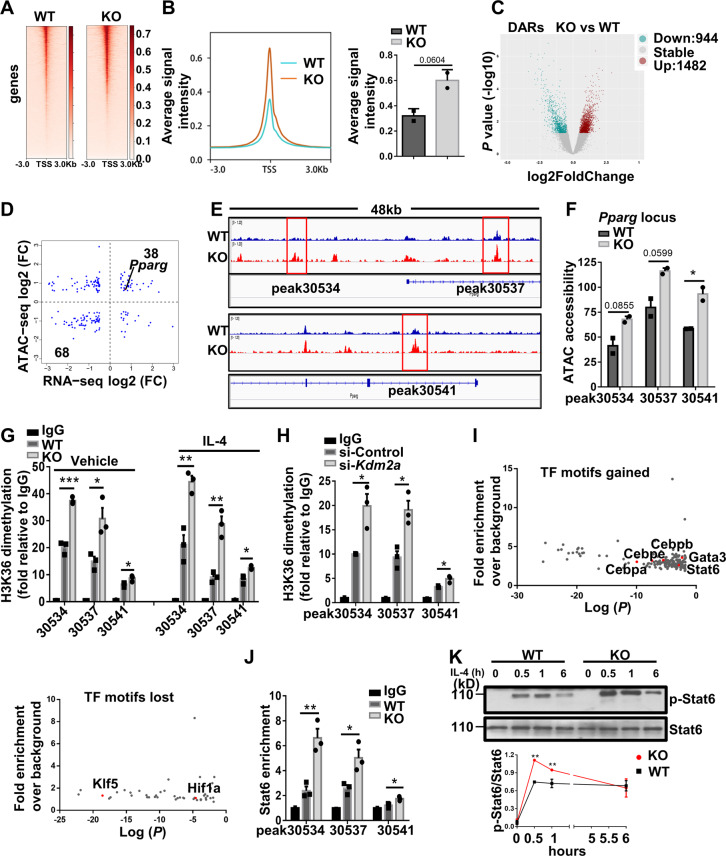
Loss of *Kdm2a* promotes Pparγ transcription by inhibiting H3K36me2 demethylation along with chromatin remodeling. **A** Average ATAC-seq signal distribution near the TSS. **B** Average ATAC-seq signals spanning the entire genome visualized in a TSS-centric manner. **C** Volcano plot of the differentially accessible regions (DARs) between WT and KO BMDMs treated with IL-4 for 6 h, as determined by ATAC-seq. DARs with log2 (fold change) ≥ 0.5, and a *P* value ≤ 0.05 in KO BMDMs are shown in red (increased accessibility) or blue (decreased accessibility). **D** Scatter plot of the correlation between DEGs vs. DARs. The number of genes in the quadrants is shown as indicated. **E** ATAC-seq bedgraph panels of the *Pparg* locus showing the peak locations relative to the TSS. The panels were compared with ATAC signals between WT and KO BMDMs following IL-4 stimulation. **F** ATAC peak strength (*y-*axis) for the selected peaks within the *Pparg* locus. **G** Results of ChIP-qPCR to compare H3K36me2 levels in the DARs of *Pparg* in WT and KO BMDMs treated with vehicle or IL-4 for 6 h. **H** Results of ChIP-qPCR to compare H3K36me2 levels in the DARs of *Pparg* in si-Control or si-*Kdm2a* transfected RAW264.7 cells after IL-4 stimulation for 6 h. **I** Enrichment of TF binding motifs in DARs of KO BMDMs gained or lost vs. WT BMDMs after IL-4 treatment. **J** ChIP-qPCR was performed for Stat6 in the *Pparg* DARs in WT and KO BMDMs treated with IL-4. **K** Representative western blot results (above) and a dotted line graph (below) showing the temporal expression of p-Stat6 in BMDMs after IL-4 stimulation. The data in (**A**–**F**) and (**I**) were derived from two independent biological replicates, and there were two mice for each biological replicate. Data are representative of three independent experiments for figures (**G**, **H**, **J**, **K**). Values are presented as mean ± SEM. Statistical significance was determined by the unpaired Student’s *t* test in (**B**, **F**–**H**, **J**, **K**). *P < 0.05; **P < 0.01.

To dissect the molecular mechanism underlying Kdm2a regulation of macrophage polarization, ATAC-seq was employed to identify the status of global chromatin accessibility. The average ATAC-seq signals around the transcription start sites (TSSs) of the entire genome were increased in the KO BMDMs as compared to the WT BMDMs (Fig. 7A, B), which is consistent with the global effect of H3K36me2 on gene transcription. Specifically, the KO BMDMs showed upregulated 1482 differentially accessible regions (DARs) predominantly located in the intergenic regions and introns, and downregulated 944 DARs that were more enriched in the intergenic regions and promoters (Figs. 7C and S6A). Given that *Pparg* was identified in the right upper quadrant of the correlation plot between DEGs and DARs (Fig. 7D), we thus assessed the changes in ATAC-seq peaks within the *Pparg* locus qualitatively, and a bedgraph was generated according to the plotted data (Fig. 7E). Exactly, *Kdm2a*^*−/−*^ led to a significant gain of peaks at the *Pparg* locus in three particular sites (Fig. 7F). Furthermore, increased openness at the *Arg1* locus was also characterized (Fig. S6B).

The next key question is whether the increased chromatin accessibility and *Pparg* transcription in the KO BMDMs were correlated to the change of H3K36me2 levels. Indeed, ChIP-PCR confirmed that the boxed peaks in Fig. 7E were enriched by H3K36me2 (Fig. S6C). Importantly, the KO BMDMs showed markedly higher enrichment than WT BMDMs, especially on peaks 30534 and 30537 (Fig. 7G), indicating that the enrichment of the *Pparg* DARs by H3K36me2 was negatively regulated by Kdm2a. The enhanced enrichment of H3K36me2 at the *Pparg* DARs was further confirmed in RAW264.7 cells using *Kdm2a* siRNAs (Figs. 7H and S6D). Since Kdm2a has been noted to influence H3K4me3 deposition [33], we also examined H3K4me3 levels on *Pparg* but failed to detect a significant difference (Fig. S6E). Next, we quantitatively assessed transcription factor binding sites where DARs were enriched. Motifs associated with the binding of canonical type 2 immunity-related transcription factors such as Stat6, C/EBPs, and Gata3 were highly enriched in the KO BMDMs (Figs. 7I and S6F). In contrast, Klf5 and Hif1a motifs were highly downregulated (Fig. 7I). To complement these in silico analyses, we directly investigated the functional relevance of the transcription factor motifs identified by ATAC-seq. ChIP-qPCR analysis indicated that *Kdm2a*^*−/−*^ promoted Stat6 recruitment, an essential factor for optimal and sustained M2 program [23], to the *Pparg* DARs (Fig. 7J). In line with the ChIP data, *Kdm2a*^*−/−*^ substantially upregulated phosphorylated Stat6 levels in BMDMs after IL-4 stimulation (Fig. 7K). Therefore, loss of *Kdm2a* facilitates active histone methylation marker H3K36me2 as well as Stat6 recruitment to the *Pparg* regulatory regions to remodel chromatin accessibility.

### *Kdm2a*^*−/−*^ orchestrates alternative activation of macrophages by targeting Pparγ

**Fig. 8:**
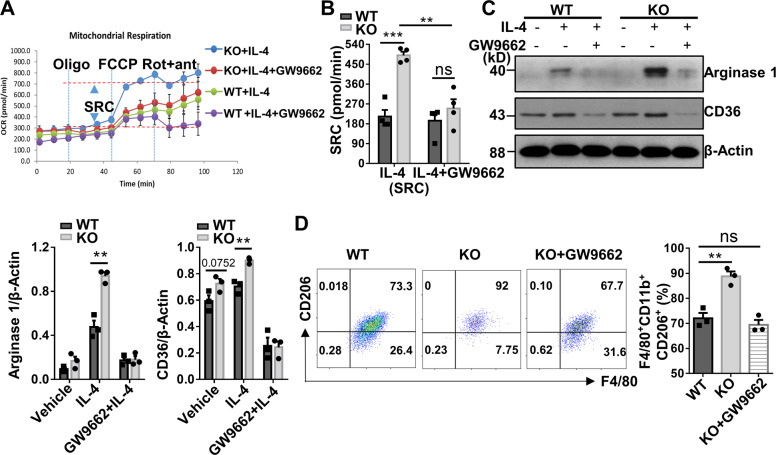
Kdm2a directly targets Pparγ to regulate alternative activation of macrophages. **A** OCR in WT and KO BMDMs stimulated with IL-4 for 24 h in the presence or absence of GW9662. **B** SRC in cells as shown in (**A**). **C** Western blot analysis of CD36 and Arg1 levels in WT and KO BMDMs under indicated culture conditions. **D** Flow cytometry analysis of the expression of F4/80, CD11b, and CD206 in BMDMs under indicated culture conditions. A bar graphic figure was employed to show the percentage of F4/80^+^CD11b^+^CD206^+^ cells in each group. Data were collected from four (**A**, **B**) or three independent experiments (**C**, **D**). Values are presented as mean ± SEM. Statistical significance was determined by the unpaired Student’s *t* test in (**C**), and by one-way ANOVA in (**B**, **D**). *P < 0.05; **P < 0.01; ***P < 0.001. ns not significant.

Since p-Stat6 has been recognized to be essential for M2 polarization [34], we first examined the direct contribution of increased p-Stat6 to M2 program in the KO macrophages by using a Stat6 inhibitor, AS1517499. AS1517499 effectively attenuated Stat6 phosphorylation (Fig. S7A), which almost abolished Arg1 expression in WT BMDMs (Fig. S7B), but a relatively high level of Arg1 expression was still noted in KO BMDMs, indicating that inhibition of p-Stat6 only partially repressed macrophage M2 program. Those data support that Pparγ, which is downstream of p-Stat6 signaling, plays a major role in modulating macrophage M2 program in *Kdm2a*^*−/−*^ mice. To address this question, GW9662, a well-established Pparγ inhibitor, was added into the BMDM cultures during IL-4 induction. Remarkably, GW9662 reduced SRC in the *Kdm2a*^*−/−*^ BMDMs to a comparative level as the WT BMDMs (Fig. 8A, B), indicating a crucial role for Pparγ in *Kdm2a* ablation-dependent metabolic reprogramming. Western blotting also demonstrated similar levels for the expression of CD36 and M2 marker, Arg1 (Fig. 8C). Furthermore, addition of GW9662 completely abolished the effect of *Kdm2a*^*−/−*^ on macrophage M2 program as evidenced by the comparative number of M2 macrophages between WT and KO BMDMs (Fig. 8D). Together, our results demonstrate that Kdm2a influences alternative activation of macrophages by targeting Pparγ.

## Discussion

In this report, we generated macrophage-specific *Kdm2a*^*−/−*^ mice by targeting the JmjC-catalytic domain. Interestingly, Kdm2a was dispensable for M1 macrophage polarization, but macrophages from *Kdm2a*^*−/−*^ mice showed elevated expression of M2 markers. A decreased *Il6* expression in the KO BMDMs was noted in a NF-κB and MAPK pathway-independent manner, which might be explained by the finding that Kdm2b, a paralog of Kdm2a, was required in macrophage for the induction of IL-6 by facilitating chromatin accessibility at the *Il6* promoter [35]. Several prior studies have addressed that the metabolic shift are required for the relevant gene expression essential for macrophage M1 or M2 programs [17, 32, 36]. Our RNA- and ATAC-seq data revealed that *Kdm2a*^*−/−*^ BMDMs were characterized by a gain of FAO signature. Indeed, the KO BMDMs manifested higher capacity to take up more fatty acids than that of WT BMDMs. The elevated fatty acid uptake and their subsequent lipolysis fueled FAO and contributed to the enhanced OXPHOS, which was further confirmed by the seahorse extracellular flux analysis, in which the KO BMDMs displayed higher basal OCR along with a markedly increased SRC in response to either vehicle or IL-4. Additionally, the increased ATAC signals at the *Pparg* locus and transcript abundance in the KO BMDMs strongly suggested that Pparγ is a major target of Kdm2a to affect alternative activation of macrophages. As expected, the enrichment of H3K36me2, which was usually associated with gene activation, at the *Pparg* locus was significantly increased in the KO BMDMs. Moreover, ATAC accessibility at Stat6, CEBPs, and Gata3 motifs was enriched in the KO BMDMs. Consistent with the changes in the accessibility at these motifs, directed ChIP analysis confirmed the occupancy of Stat6 at the *Pparg* DARs in the KO BMDMs in the presence of IL-4. Notably, inhibition of Pparγ almost completely attenuated the upregulated M2 macrophage signature in the KO BMDMs, indicating that *Kdm2a*^*−/−*^ promotes M2 program predominantly by upregulating Pparγ expression, although we cannot completely rule out that additional mechanisms might be involved.

There are two main types of adipose tissues, WAT and BAT. WAT mainly stores energy and is increased in obesity, whereas BAT dissipates energy via specific expression of Ucp1 [37]. In addition to “classic BAT,” some white fat cells, particularly in the subcutaneous fat depot, can also differentiate into beige adipocytes in response to diverse stimuli including cold exposure [38]. Since the initial description of WAT being infiltrated by macrophages in obese animals [30], numerous studies have shown that M1 macrophages promote adipocyte dysfunction and potentiate insulin resistance, while M2 macrophages enhance the activation of BAT and beiging of WAT, thereby modulating adaptive thermogenesis and energy expenditure [39–41]. Given that loss of *Kdm2a* orchestrated alternative activation of macrophages, we thus used KO mice to dissect the impact of histone methylation on the pathoetiology of obesity. Our results demonstrated that mice deficient in myeloid *Kdm2a* are protected from HFD-induced obesity, insulin resistance, and hepatic steatosis. These results are reminiscent of the phenotype in *LysM-*Cre-*Pparg*^f/f^ mice [42]. Moreover, the metabolic phenotypes largely attributed to the changes in ATMs, including the alleviated macrophage accumulation and enhanced M2 program in both epWAT and scWAT. Reversed M1–M2 imbalance of ATMs contributed to the maintenance of energy homeostasis in mice lacking *Kdm2a*, as evidenced by the increase of RER, food intake, and expression of genes associated with energy expenditure following HFD challenge. In response to cold exposure, abrogation of myeloid *Kdm2a* in mice resulted in particularly robust augmentation of the thermogenic activity of both brown and beige fat, further validated that myeloid *Kdm2a* ablation promotes adaptive thermogenesis.

Our findings also raised additional issues that are worthy of further investigations. First, the mechanisms underlying *Kdm2a* deficiency induction of higher p-Stat6 levels following IL-4 stimulation merit in-depth study. IL-4 stimulation triggers JAK-Stat6 activation, thereby transcribing the expression of genes essential for the macrophage M2 program. JAK kinases are well known as ATP-dependent kinases and phosphorylate Stat6 by catalyzing the transfer of the γ-phosphate from ATP to Stat6, and therefore, alterations in ATP production may influence the phosphorylation of Stat6 [43]. We thus hypothesize that loss of *Kdm2a* promotes macrophage M2 programming by regulating a positive feedback loop, in which *Kdm2a* deficiency upregulates Pparγ through facilitating H3K36me2 levels, and Pparγ, in turn, promotes OXPHOS to generate more ATP, which then enhances Stat6 phosphorylation to further transcribe Pparγ. However, this hypothesis need to be further explored in the following studies. Second, a recent study demonstrated evidence that NSD2 overexpression in multiple myeloma opens the chromatin through deposition of H3K36me2, which allows binding of transcription factors such as AP-1, to recruit CBP/p300, thereby mediating the deposition of H3K27ac, a well-defined marker of enhancer activity [44]. It would be, therefore, plausible to explore whether H3K36me2 provides a favorable environment for the H3K27ac enrichment to regulate gene transcription synergistically. Third, it has now been recognized that M2 macrophages can be therapeutically exploited to treat obesity [45], but additional studies would be necessary to translate our current discoveries into clinical settings, particularly for the development of Kdm2a inhibitors with minimal side effect. Furthermore, the Pparγ agonists, such as thiazolidinediones, are potent to activate Pparγ with robust insulin-sensitizing effect [46]. However, a wide array of deleterious side effects, such as weight gain, fluid retention, and osteoporosis, limited their clinical applications. As such, developing a novel drug delivery system that can specifically target macrophages might help to overcome those issues, which may also apply to the development of Kdm2a inhibitors as well.

In summary, the present study provided evidence demonstrating that loss of *Kdm2a* promotes macrophage M2 program, which involves enhanced chromatin accessibility at the *Pparg* locus associated with increased H3K36me2 levels. This epigenetic change resulted in enhanced fatty acid uptake and metabolic reprogramming. As a result, mice deficient in myeloid *Kdm2a* were protected from HFD-induced obesity and insulin resistance. Those results expanded our understanding of the cross talk between immune cells and adipose tissue, indicating that Kdm2a could be a viable epigenetic target for developing more efficacious and cost-effective therapies for the prevention and treatment of obesity and insulin resistance in clinical settings.

## Supplementary information

Supplementary Information

Supplemental Figure 1

Supplemental Figure 2

Supplemental Figure 3

Supplemental Figure 4

Supplemental Figure 5

Supplemental Figure 6

Supplemental Figure 7

## Data Availability

All data needed to evaluate the conclusions in the paper are present in the paper and/or Supplementary information. Additional data related to this paper may be requested from the authors.
