# Supplementary material for: Kdm2a deficiency in macrophages enhances thermogenesis to protect mice against HFD-induced obesity by enhancing H3K36me2 at the Pparg locus
Source: Cell Death Differ. 2021 Jan 18;28(6):1880–99. doi: 10.1038/s41418-020-00714-7 (PMC8185071; doi:10.1038/s41418-020-00714-7)
Supplement: Supplementary file 1 — Supplementary Information [file 41418_2020_714_MOESM1_ESM.docx]

Supplementary Information for

***Kdm2a* deficiency in macrophages enhances thermogenesis to protect mice against HFD-induced obesity by enhancing H3K36me2 at the *Pparg* locus**

Longmin Chen^1,†^, Jing Zhang^1,†^, Yuan Zou^1^, Faxi Wang^1^, Jingyi Li^1^, Fei Sun^1^, Xi Luo^1^, Meng Zhang^1,2^, Yanchao Guo^1,2^, Qilin Yu^1^, Ping Yang^1^, Qing Zhou^1^, Zhishui Chen^1,3^, Huilan Zhang^1^, Quan Gong^4^, Jiajun Zhao^5^, Decio L. Eizirik^6^, Zhiguang Zhou^7^, Fei Xiong^1,*^, Shu Zhang^1,*^ and Cong-Yi Wang^1,*^

^†^These authors contributed equally to this work.

*Corresponding authors: Cong-Yi Wang (Tel: 86-27-6937-8458; email: [wangcy@tjh.tjmu.edu.cn](mailto:wangcy@tjh.tjmu.edu.cn)) or Shu Zhang (email: szhang@tjh.tjmu.edu.cn), or Fei Xiong (email: [feixiong@tjh.tjmu.edu.cn](mailto:feixiong@tjh.tjmu.edu.cn))

The file includes:

Fig. S1. H3K36me2 status in CD45^-^ SVF and splenocytes during obesity.

Fig. S2. Generation of macrophage-specific *Kdm2a* deficient mice.

Fig. S3. Loss of *Kdm2a* neither inﬂuences the development and maturation of myeloid cells nor aﬀects the activation of peripheral lymphoid cells.

Fig. S4. *Kdm2a* deficiency does not affect NF-κB and MAPK signaling pathways and only affects a few transcriptional factors relevant to macrophage polarization.

Fig. S5. Comparison of metabolic phenotyping between WT and KO mice.

Fig. S6. Genomic distribution of DARs and binding consensus of the TFs.

Fig. S7. Effect of upregulated p-Stat6 on enhanced M2 program in KO BMDMs.

Table S1. Primer sequences for real-time PCR

Table S2. Primer sequences for ChIP-qPCR

**Fig. S1 H3K36me2 status in CD45^-^ SVF and splenocytes during obesity.** (A and B) Flow cytometry analysis of H3K36me2 status in CD45^-^ SVF from epWAT (A) and scWAT (B) of mice after 8 weeks of HFD or ND feeding. (C and D) Flow cytometry analysis of H3K36me2 status in splenic CD45^+^ cells (C) and macrophages (D) in mice after 8 weeks of HFD or ND feeding. Left: a representative flow cytometry data; right: quantitative data of analyzed in all mice. n = 5 per group. Values are expressed as mean ± SEM; unpaired Student’s *t* test.

**Fig. S2 Generation of macrophage-specific *Kdm2a* deficient mice.** (A) KO mice were identified by PCR genotyping. (B) Representative Western blot analysis and quantification of Kdm2a and H3K36me2 levels in CD4^+^ T cells and different tissues from WT and KO mice. (C) mRNA levels of *Kdm2b* in WT and KO BMDMs as measured by RT-PCR. Data are presented as mean ± SEM of 3 independent biological replicates.

**Fig. S3 Loss of *Kdm2a* neither inﬂuences the development and maturation of myeloid cells nor aﬀects the activation of peripheral lymphoid cells.** (A-D) Representative FACS plots of CD11b^+^F4/80^+^ macrophages (Ma), CD11b^+^Gr-1^+^ neutrophils (Neu), total CD11c^+^ DCs (DCs), CD11c^+^B220^-^ conventional dendritic cells (cDCs), and CD11c^+^B220^+^ plasmacytoid dendritic cells (pDCs) in bone marrow (A and B) and in spleen (C and D) from WT and KO mice under steady state. (E and F) Summary graphs showing the cell numbers of indicated subsets as in (A-D). (G-I) Representative FACS plots of total CD4^+^ and CD8^+^ T cells, and CD4^+^Foxp3^+^ regulatory T cells (Tregs) in spleen (G), mesenteric lymph node (MLN; H), and inguinal lymph node (ILN; I) from WT and KO mice. (J) Total cellularity of the spleen, MLN and ILN. (K-M) Frequencies of total CD4^+^ and CD8^+^ T cells, CD4^+^ and CD8^+^ effector memory (CD62L^low^CD44^hi^) and naive (CD62L^hi^CD44^low^) T cells, and Tregs in spleen (K), MLN (L) and ILN (M) from WT and KO mice. In (A-M), 8-week-old male mice were used. n = 4 per genotype. Values shown are mean ± SEM; unpaired Student’s *t* test.

**Fig. S4. *Kdm2a* deficiency does not affect NF-κB and MAPK signaling pathways and only affects a few transcriptional factors relevant to macrophage polarization.** (A) Representative Western blot results (left) and the line graphs (right) showing temporal expression of the following proteins in BMDMs stimulated with LPS for indicated time: p-P65, IκBα, p-JNK and p-P38. (B) Flow cytometry analysis of the expression of F4/80, CD11b and CD206 in BMDMs without IL-4 treatment. Percentages of F4/80^+^CD11b^+^CD206^+^ cells are shown. (C) Expression of macrophage polarization-related genes by WT and KO BMDMs upon IL-4 stimulation. The names of the genes that were differentially expressed in the two genotypes are highlighted in red. Data are representative of 2 (A) or 3 (B) independent experiments. Values represent mean ± SEM; unpaired Student’s *t* test.

**Fig. S5 Comparison of metabolic phenotyping between WT and KO mice.** (A) Comparison of blood glucose levels between WT and KO mice under fasting condition. (B) Results of intraperitoneal glucose tolerance tests (left) and the areas under curves (AUC) for blood glucose levels (right) at 16 weeks of ND induction. (C) Results for the intraperitoneal insulin tolerance tests (left) and areas above curves (AAC) at 16 weeks of ND induction. (D and E) Representative FACS plots and quantitative analysis of H3K36me2 status in ATMs in epWAT (D) and scWAT (E) from mice after 8 weeks of HFD or ND feeding. n = 3 per group. (F) RER monitored over a 24-h period (left) and shown as averaged values (right). (G) Analysis of food intake (g) of ND-fed WT and KO mice by metabolic cages. In (A-C, F and G), male KO mice and age-matched WT littermate were fed a ND for 16 weeks (n = 4 for each group). Values are presented as mean ± SEM. Significance was determined by unpaired Student's *t* test in A-C and F and by one-way ANOVA in D and E. **P* < 0.05; ***P* < 0.01. ns, not significant.

**Fig. S6. Genomic distribution of DARs and binding consensus of the TFs**. (A) Genomic distribution of regions with increased (left) and decreased (right) chromatin accessibility, as measured by ATAC-seq. (B) ATAC-seq bedgraph panels of the *Arg1* locus showing the chromatin accessibility levels in WT and KO BMDMs with IL-4. (C) ChIP-PCR results for analysis of H3K36me2 enrichment in the *Pparg* DARs in BMDMs. (D) Verification of the efficiency of siRNA against *Kdm2a* at the protein level. (E) Results of ChIP-qPCR to compare H3K4me3 modification in the DARs of *Pparg* in WT and KO BMDMs following IL-4 stimulation for 6 hours. (F) Binding consensus of Stat6, C/EBPb and Gata3 in BMDMs. (A, B and F) are from 2 independent biological replicates. Data in (E) are representative of 3 independent experiments. Values are presented as mean ± SEM; unpaired Student's *t* test.

**Fig. S7 Effect of upregulated p-Stat6 on enhanced M2 program in KO BMDMs.** (A) Inhibition efficiency of AS1517499 on Stat6 phosphorylation. (B) Western blot analysis of Arginase 1 levels in WT and KO BMDMs under indicated culture conditions. Data are representative of 3 independent experiments. Values represent mean ± SEM. Significance was determined by unpaired Student's *t* test. **P* < 0.05.

**Table S1. Primer sequences for real-time PCR**

| Gene | Forward (5’-3’) | Reverse (5’-3’) |
| --- | --- | --- |
| *Ucp1* | AGGCTTCCAGTACCATTAGGT | CTGAGTGAGGCAAAGCTGATTT |
| *Cox5a* | GGAAGACCCTAATCTAGTCCCG | GTTGGGGCATCGCTGACTC |
| *Cox7a* | GCTCTGGTCCGGTCTTTTAGC | GTACTGGGAGGTCATTGTCGG |
| *Cox8b* | TGTGGGGATCTCAGCCATAGT | AGTGGGCTAAGACCCATCCTG |
| *Pparg* | TCATGACCAGGGAGTTCCTC | CAGGTTGTCTTGGATGTCCTC |
| *Cd36* | TCATGCCAGTCGGAGACATGCTTA | AACTGTCTGTACACAGTGGTGCCT |
| *Nos2* | ACATCGACCCGTCCACAGTAT | CAGAGGGGTAGGCTTGTCTC |
| *Il6* | ATGGATGCTACCAAACTGGAT | TGAAGGACTCTGGCTTTGTCT |
| *Tnf* | ACTGAACTTCGGGGTGATCG | GGCTACAGGCTTGTCACTCG |
| *Actin* | AGAGGGAAATCGTGCGTGAC | CAATAGTGATGACCTGGCCGT |
| *Arg1* | CTCCAAGCCAAAGTCCTTAGAG | AGGAGCTGTCATTAGGGACATC |
| *Retnla* | CTGGGTTCTCCACCTCTTCA | TGCTGGGATGACTGCTACTG |
| *Mrc1* | CTCTGTTCAGCTATTGGACGC | CGGAATTTCTGGGATTCAGCTTC |
| *Il1b* | TACGGACCCCAAAAGATGA | TGCTGCTGCGAGATTTGAAG |
| *Lpl* | GGGAGTTTGGCTCCAGAGTTT | TGTGTCTTCAGGGGTCCTTAG |
| *Plin3* | ATGTCTAGCAATGGTACAGATGC | CGTGGAACTGATAAGAGGCAGG |
| *Ccl2* | TTAAAAACCTGGATCGGAACCAA | GCATTAGCTTCAGATTTACGGGT |

**Table S2. Primer sequences for ChIP-qPCR**

| Site | Forward (5’-3’) | Reverse (5’-3’) |
| --- | --- | --- |
| *Pparg*-30534 | GTTTGTCCCACATTAGCAACTTTTG | AACTTTTAAGCAGGGAGAAAGTTTTGCAG |
| *Pparg*-30537 | CACACACCCACACTCTGTAGTTCTC | GGGTAATCACATAAGGTCTTATTC |
| *Pparg*-30541 | GCTCCTACCTACACTGTGCATGAGAT | CCAGCTCTCTGGTTTTTAGTGTTTAGG |
