# Supplementary figures and images for: Kdm2a deficiency in macrophages enhances thermogenesis to protect mice against HFD-induced obesity by enhancing H3K36me2 at the Pparg locus
Source: Cell Death Differ. 2021 Jan 18;28(6):1880–99. doi: 10.1038/s41418-020-00714-7 (PMC8185071; doi:10.1038/s41418-020-00714-7)

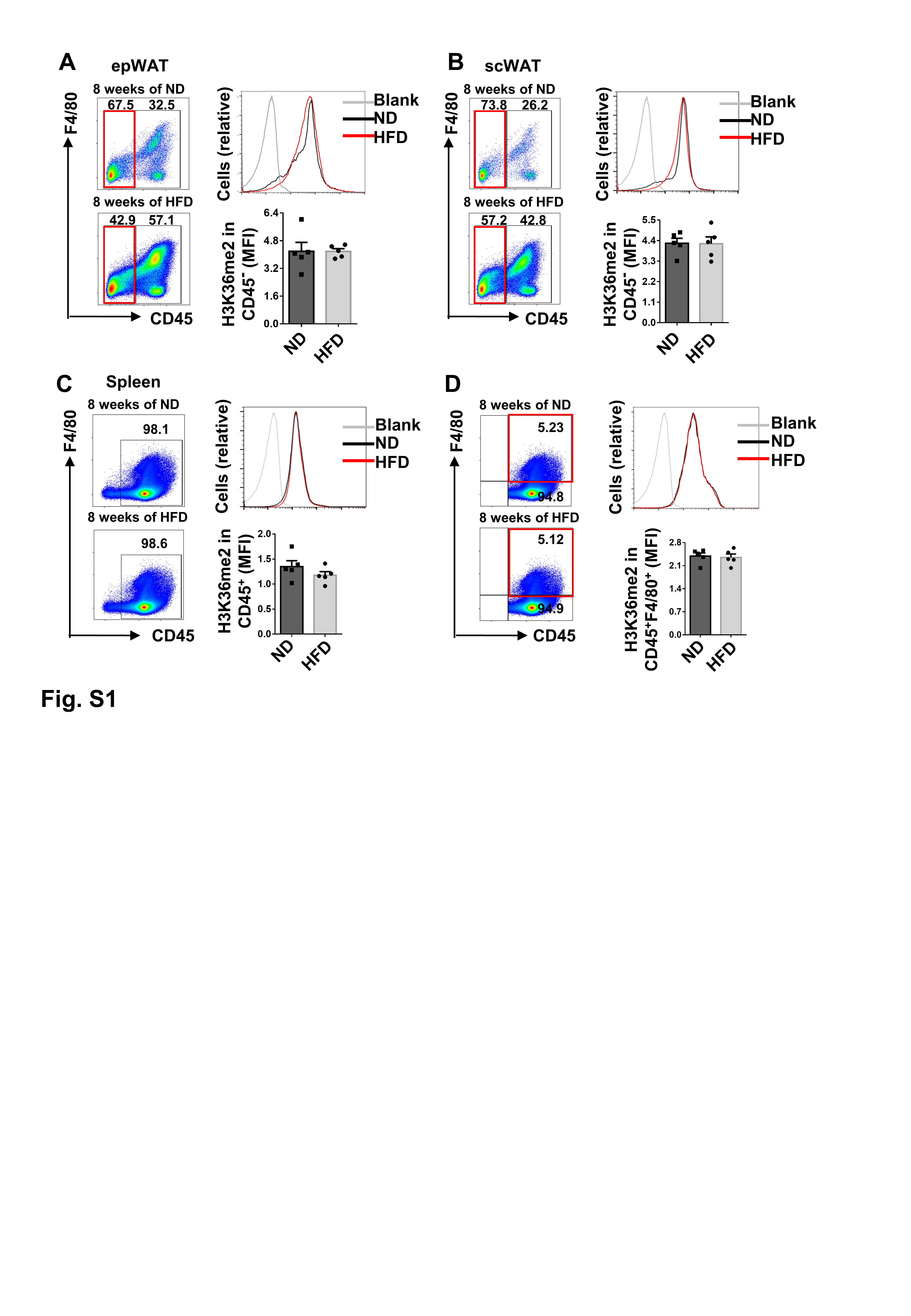

Supplement: Supplementary file 2 — Supplemental Figure 1 [file 41418_2020_714_MOESM2_ESM.tif]

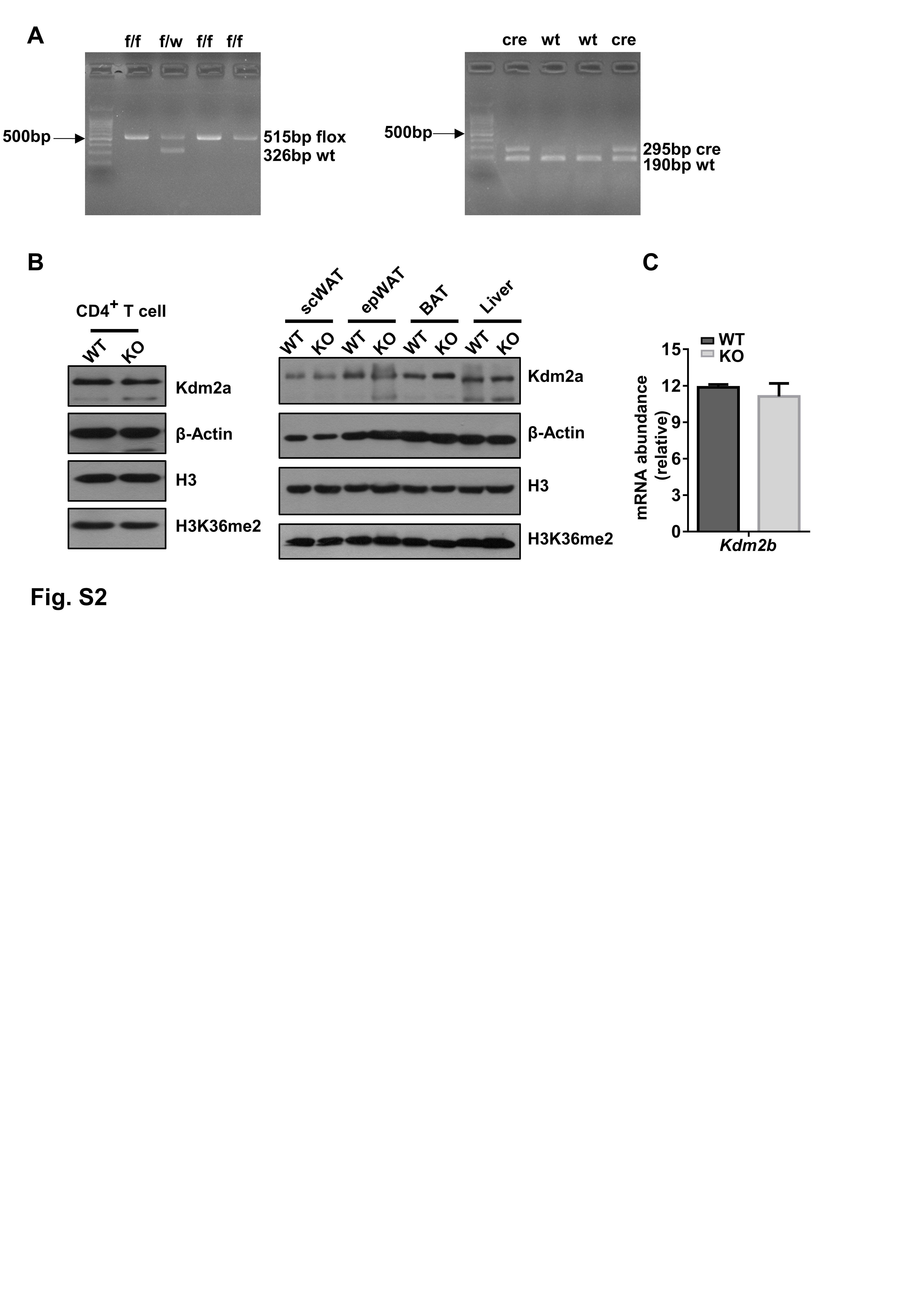

Supplement: Supplementary file 3 — Supplemental Figure 2 [file 41418_2020_714_MOESM3_ESM.tif]

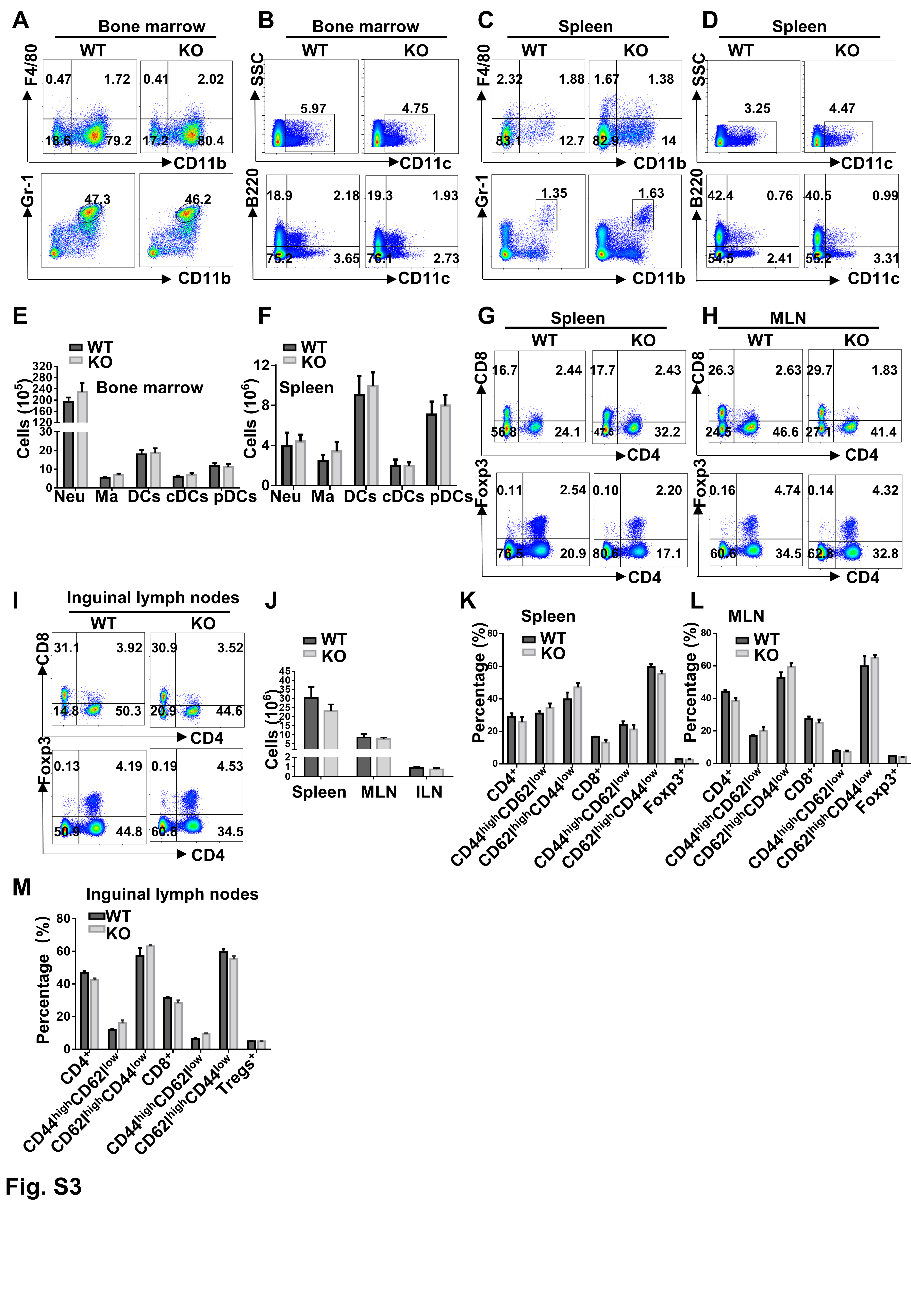

Supplement: Supplementary file 4 — Supplemental Figure 3 [file 41418_2020_714_MOESM4_ESM.tif]

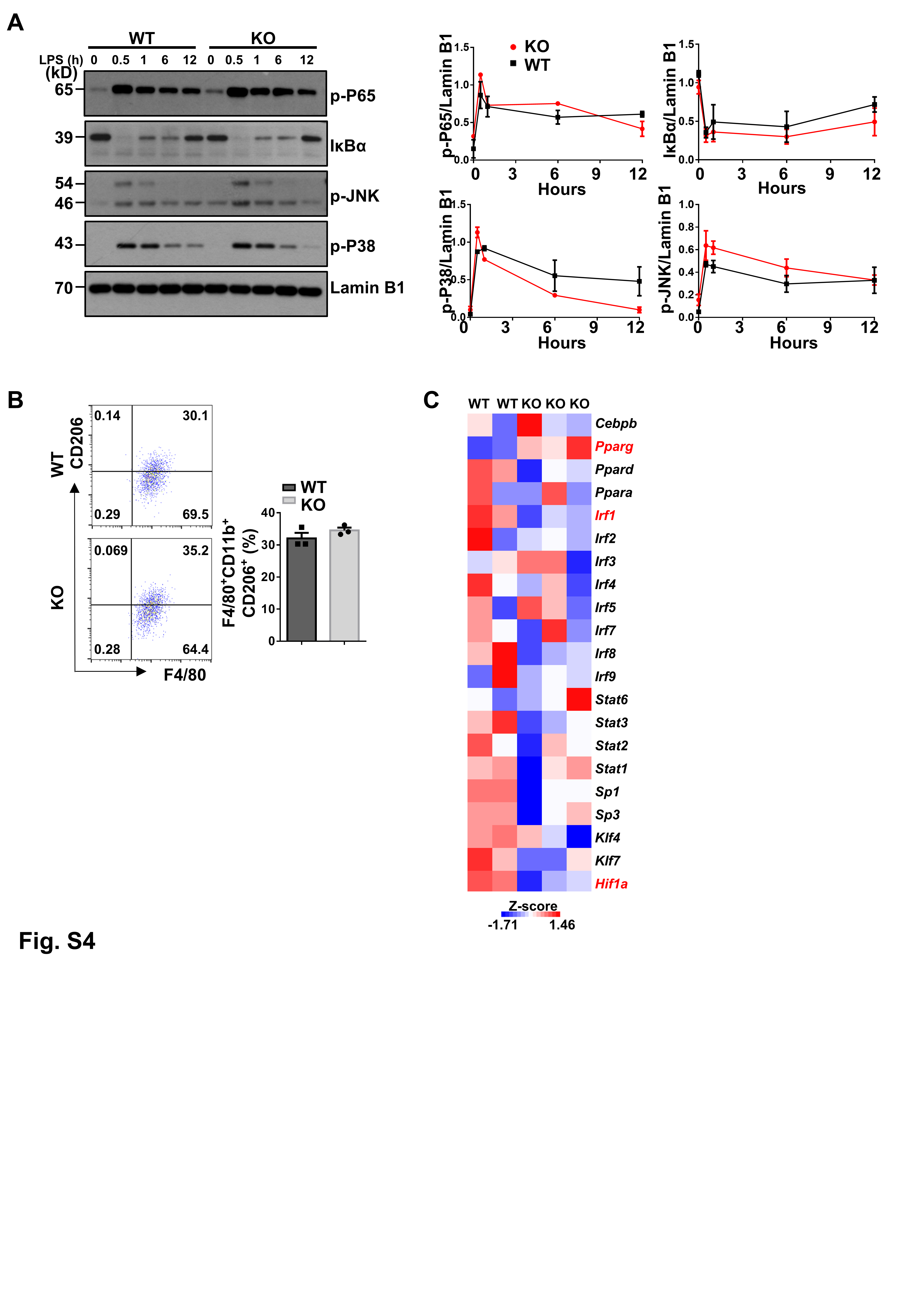

Supplement: Supplementary file 5 — Supplemental Figure 4 [file 41418_2020_714_MOESM5_ESM.tif]

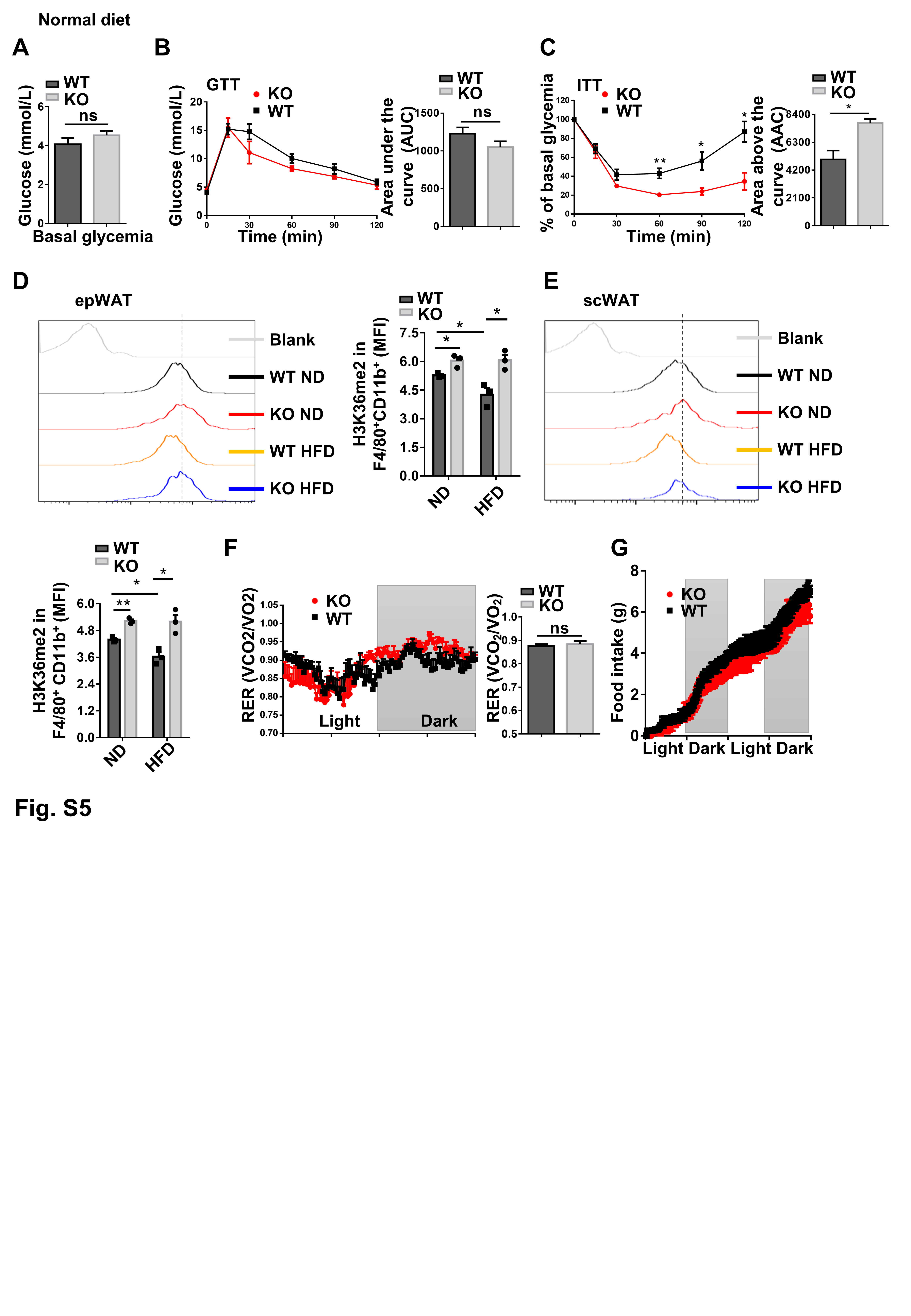

Supplement: Supplementary file 6 — Supplemental Figure 5 [file 41418_2020_714_MOESM6_ESM.tif]

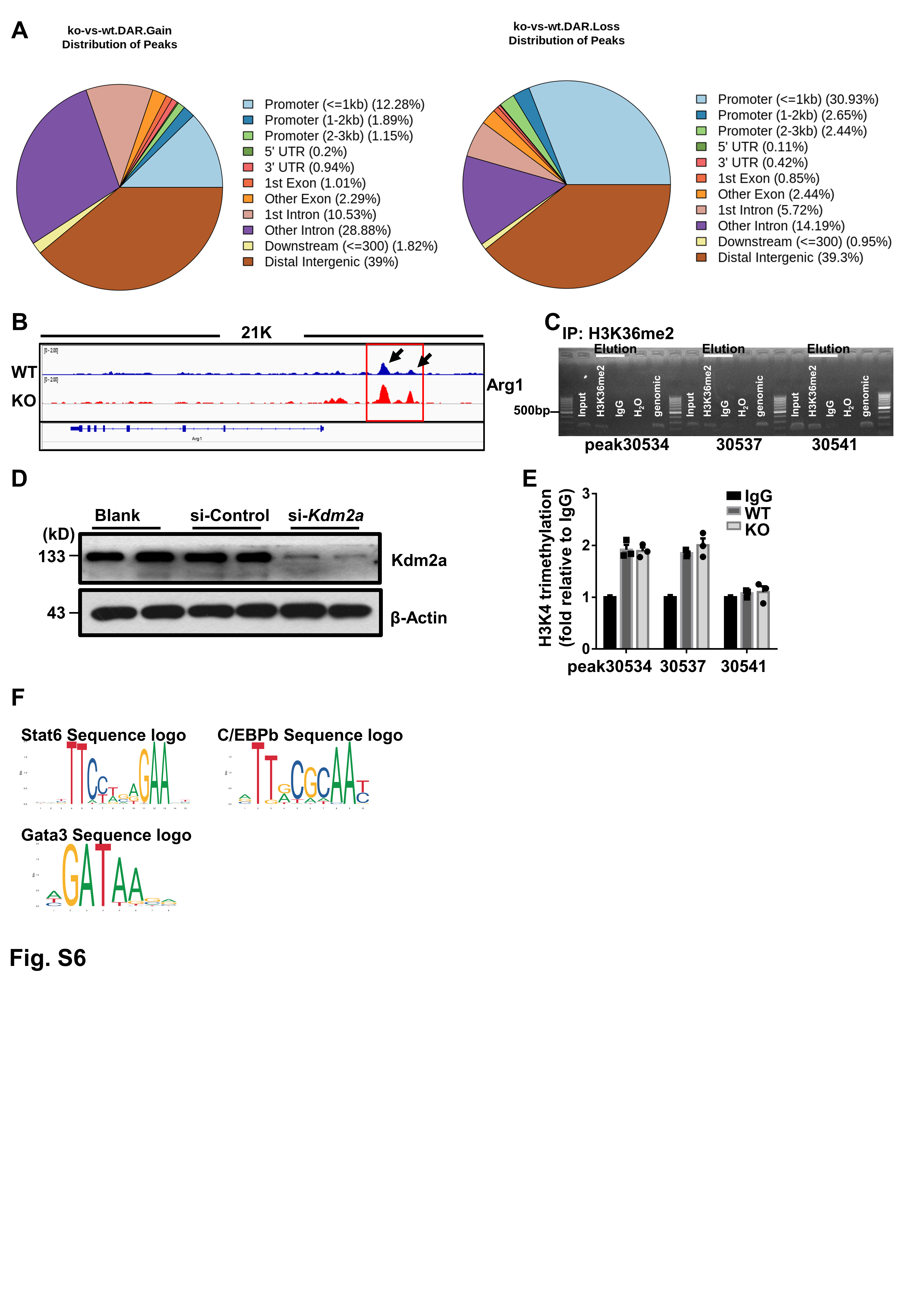

Supplement: Supplementary file 7 — Supplemental Figure 6 [file 41418_2020_714_MOESM7_ESM.tif]

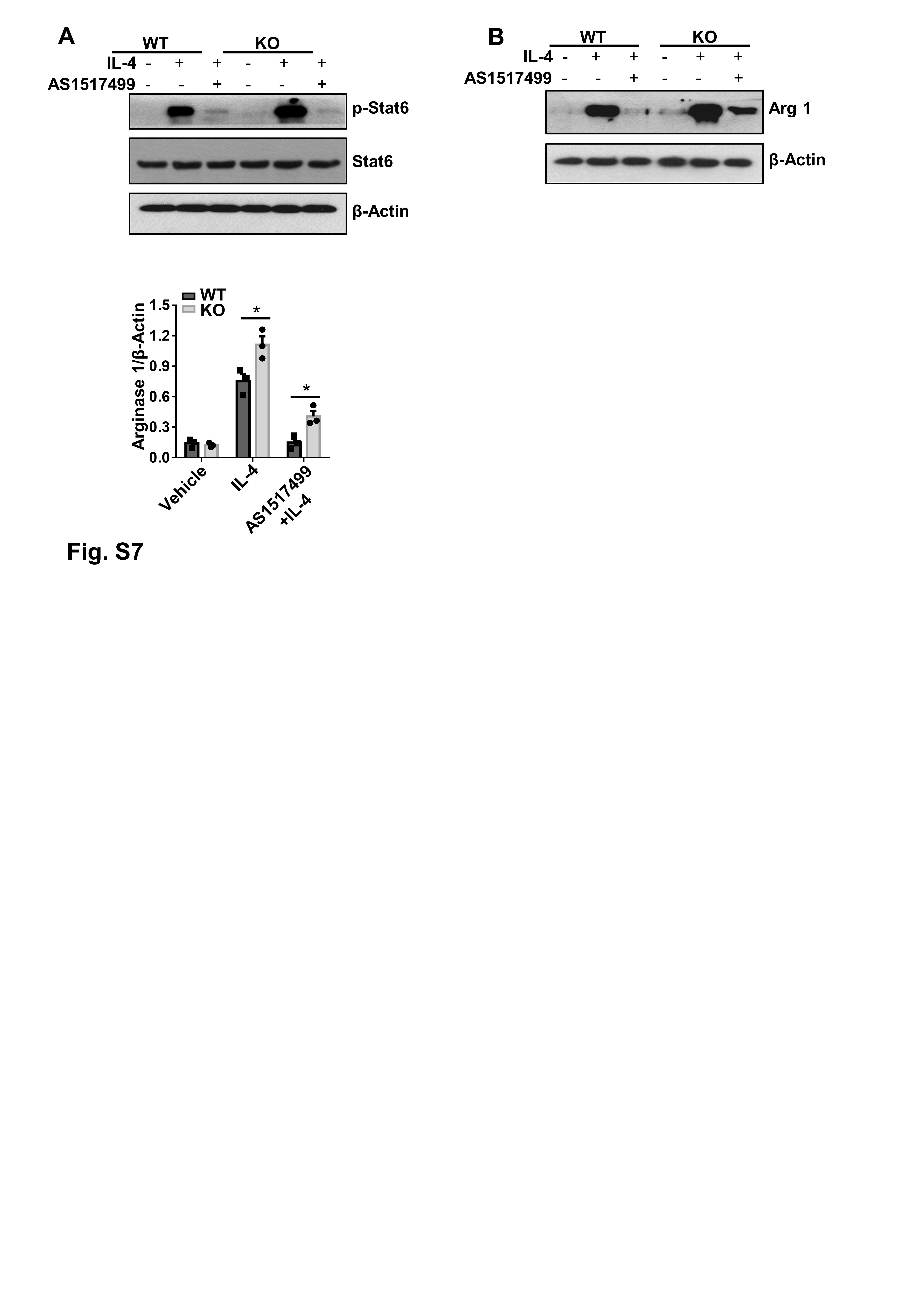

Supplement: Supplementary file 8 — Supplemental Figure 7 [file 41418_2020_714_MOESM8_ESM.tif]
